# Magnetic Anomaly Detection Based on a Multi-Parameter-Constrained Mirror Dual-Branch Biased Monostable Stochastic Resonance System

**DOI:** 10.3390/s26123776

**Published:** 2026-06-13

**Authors:** Rongxiang Xia, Mingxi Chen, Lizhi Hong, Zhiyuan Ai, Shaojie Ma

**Affiliations:** School of Mechanical Engineering, Nanjing University of Science and Technology, Nanjing 210094, China; xrx@njust.edu.cn (R.X.); chenmx@njust.edu.cn (M.C.); h-relife@njust.edu.cn (L.H.); aizhiyuan1996@njust.edu.cn (Z.A.)

**Keywords:** magnetic anomaly detection, stochastic resonance, biased monostable system, mirror dual-branch structure, robust Bayesian optimization

## Abstract

Magnetic anomaly detection is vulnerable to environmental noise and insufficient prior target information, making non-periodic anomaly signals difficult to detect at low-signal-to-noise-ratio (SNR) conditions. This paper proposes a detection method based on a multi-parameter-constrained mirror dual-branch biased monostable stochastic resonance (SR) system. Nonlinear odd-order bias terms are introduced into the conventional biased monostable potential function to build a multi-parameter-controllable SR model. This improves regulation of potential-well width, depth, and wall morphology, enhancing noise-energy utilization and responses to non-periodic features. Considering peak-type, valley-type, and bipolar anomaly morphologies, a mirror dual-branch SR structure is developed to cooperatively detect features with different polarities. To preserve temporal waveforms and time–frequency structures during parameter optimization, a composite metric combining the correlation coefficient and wavelet-domain image structural similarity index is constructed. Multi-fidelity robust Bayesian optimization is used to obtain a unified robust parameter set for the magnetic anomaly signal family. Experiments with simulated colored noise and measured geomagnetic noise show that the proposed method effectively recovers magnetic anomaly features under strong noise. At −19 dB SNR, its detection probability remains above 80%. Compared with orthogonal basis function decomposition, empirical mode decomposition, and complete ensemble empirical mode decomposition with adaptive noise, the method achieves better noise suppression, feature preservation, and detection performance under low-SNR conditions.

## 1. Introduction

Ferromagnetic targets in the geomagnetic field can induce local magnetic-field distortions, which provide the physical basis for magnetic anomaly detection (MAD). Because MAD is usually implemented in a passive sensing manner, it offers advantages such as good concealment and weak dependence on target emission characteristics. Therefore, it has been widely used in public safety inspection, unexploded ordnance detection, nondestructive testing, geophysical exploration, and bio-magnetic measurement [1,2,3,4,5]. However, in practical engineering environments, magnetic anomaly signals are usually weak, short-duration, non-periodic, and morphologically variable. They are often submerged by complex environmental noise, sensor-intrinsic noise, and front-end circuit noise. Therefore, magnetic anomaly detection under low-signal-to-noise-ratio (SNR) conditions remains a challenging problem.

Existing MAD methods can be broadly divided into two categories: target-prior-model-based methods and non-parametric methods based on noise statistical characteristics. The former usually rely on signal representation methods such as dipole models, orthogonal basis function decomposition [6], matched filtering [7], wavelet matched filtering [8], or principal component analysis [9]. These methods can achieve good detection performance when the target model and observation geometry are clearly defined. However, when prior information such as the target magnetic moment direction, encounter angle, encounter distance, or target morphology is unknown, the actual magnetic anomaly signal may deviate significantly from the predefined template, leading to performance degradation in matched-filtering-based methods. The latter category performs detection using minimum entropy detection [10], higher-order crossing statistics [11], or other statistical features, thereby reducing dependence on an accurate target model. Nevertheless, under extremely low-SNR conditions or strong colored-noise backgrounds, these methods still suffer from insufficient detection stability. In recent years, deep learning methods have also been applied to MAD [12,13]. However, they usually require a large number of labeled samples, complex preprocessing, and considerable computational resources, which limits their applicability in small-sample and real-time detection scenarios.

Stochastic resonance (SR) provides an alternative approach for weak non-periodic signal detection. Through the cooperative interaction among a nonlinear system, the input signal, and noise, SR allows part of the noise energy to contribute to the dynamical enhancement of weak signal responses [14]. Unlike conventional linear filtering, SR does not simply suppress noise; instead, it exploits noise of appropriate intensity to improve the observability of weak signals. Classical bistable and multi-stable SR systems have been widely studied for periodic weak-signal detection, but their mechanisms usually rely on noise-assisted transitions of particles across potential barriers [15]. However, output saturation in classical bistable and multi-stable SR systems may limit the further enhancement in weak signals. To alleviate this problem, piecewise unsaturated potential functions [16], continuous unsaturated potential functions [17], and periodic potential functions [18] have been introduced into SR systems to improve the potential-function structure and reduce the influence of output saturation. These studies indicate that potential-function design plays an important role in the weak-signal enhancement performance of SR systems.

Nevertheless, for a single pulse or a non-periodic magnetic anomaly signal, an enhancement mechanism dominated by inter-well transitions may make the system output resemble a triggering event rather than the original waveform, resulting in the loss of morphological information. In contrast, a monostable SR system has no conventional inter-well potential barrier [19] and can maintain a continuous dynamic response to input perturbations within a single potential well. Therefore, monostable and single-well SR mechanisms provide a more suitable framework for preserving the local time-domain features of non-periodic signals. Related studies have shown that single-well or biased monostable SR systems can be used for feature extraction and recovery of non-periodic signals under noisy conditions [20,21].

Although biased monostable SR shows promising potential for weak non-periodic signal detection, two key issues remain. First, conventional biased monostable potential functions have limited degrees of freedom, making it difficult to simultaneously regulate the potential-well depth, width, and wall morphology. As a result, their adaptability to magnetic anomaly signals with different waveform characteristics is insufficient. Second, the performance of an SR system depends strongly on parameter selection, while a single evaluation metric is usually unable to characterize both time-domain waveform similarity and time–frequency structural similarity. This may lead to optimized parameters that are effective only for a specific signal type or noise condition. For a magnetic anomaly signal family generated by different encounter angles and magnetic moment directions, a more reasonable strategy is not to optimize parameters for a single sample, but to obtain a unified robust parameter set with good generalization ability across multiple signal morphologies.

To address these problems, this paper proposes a multi-parameter-constrained mirror dual-branch biased monostable SR method. The main contributions of this study are as follows: (1) a multi-parameter-constrained biased monostable potential function is proposed, in which nonlinear odd-order bias terms are introduced to enhance potential-shape controllability; (2) a mirror dual-branch SR detection structure is constructed to simultaneously respond to peak-type, valley-type, and bipolar magnetic anomaly signals; (3) a composite evaluation metric combining the correlation coefficient and the wavelet-domain image structural similarity index is proposed to jointly constrain time-domain waveform preservation and time–frequency structural recovery; (4) a multi-fidelity robust Bayesian optimization strategy is designed to obtain a unified robust parameter set for the magnetic anomaly signal family; and (5) experiments with simulated colored noise and measured geomagnetic noise are conducted to verify the detection advantages of the proposed method under low-SNR conditions.

## 2. Theoretical Background

### 2.1. Magnetic Anomaly Signal Model

In magnetic anomaly detection, a ferromagnetic target can generally be approximated as a magnetic dipole when the observation distance between the magnetic sensor and the target is significantly larger than the characteristic size of the target. This approximation is commonly considered applicable when the observation distance exceeds approximately three times the maximum target dimension. Let the equivalent magnetic moment of the ferromagnetic target be $M=[m_{x},m_{y},m_{z}{]}^{T}$, and let $r=[x,y,z{]}^{T}$ denote the position vector from the magnetic dipole to the observation point. The magnetic anomaly field generated by the target at the observation point can be expressed as

(1) $$B_{r}=\frac{\mu_{0}}{4\pi }\left[\frac{3(M\cdot r)}{{\left|r\right|}^{5}}-\frac{M}{{\left|r\right|}^{3}}\right]$$

where $\mu_{0}$ is the permeability of free space, M is the equivalent magnetic moment of the target, r is the position vector from the dipole to the observation point, and |r| denotes the observation distance.

**Figure 1:**
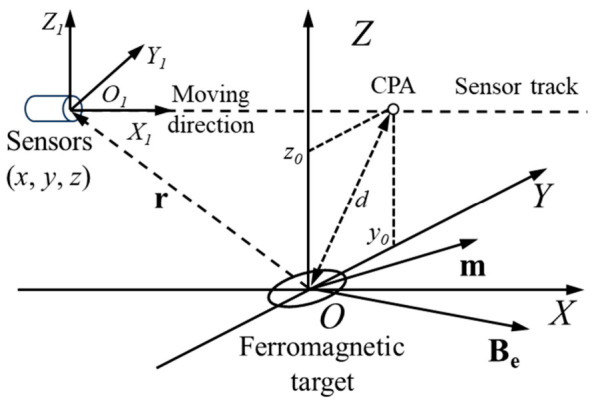
Schematic diagram of the geometric model for magnetic anomaly detection.

Figure 1 illustrates the geometric model used for magnetic anomaly detection in this study. For theoretical derivation, the magnetic dipole is placed at the origin of a Cartesian coordinate system, and the magnetic sensor is assumed to move along the positive X-axis. For a vector magnetic sensor, the total magnetic field measured at the observation point can be expressed as the superposition of the background geomagnetic field $B_{e}$ and the target-induced anomaly field $B_{r}$, namely $B_{t}=B_{e}+B_{r}$. Within a short observation window, the background geomagnetic field usually varies slowly and can therefore be approximated as a constant vector. After removal of the background field, the magnetic anomaly vector at the observation point can be written as

(2) $$\begin{array}{c} \Delta B=B_{r}=\left[\begin{array}{c} B_{\mathrm{rx}}\\ B_{\mathrm{ry}}\\ B_{\mathrm{rz}} \end{array}\right]=\frac{\mu_{0}}{4\pi |r{|}^{5}}\left[\begin{array}{ccc} 2x^{2}-y^{2}-z^{2} & 3xy & 3xz\\ 3xy & 2y^{2}-x^{2}-z^{2} & 3yz\\ 3xz & 3yz & 2z^{2}-x^{2}-y^{2} \end{array}\right]\left[\begin{array}{c} m_{x}\\ m_{y}\\ m_{z} \end{array}\right]\\ =\frac{\mu_{0}}{4\pi {\left(x^{2}+y^{2}+z^{2}\right)}^{5/2}}\left[\begin{array}{c} (2x^{2}-y^{2}-z^{2})m_{x}+3xym_{y}+3xzm_{z}\\ (2y^{2}-x^{2}-z^{2})m_{y}+3xym_{x}+3yzm_{z}\\ (2z^{2}-x^{2}-y^{2})m_{z}+3xzm_{x}+3yzm_{y} \end{array}\right] \end{array}$$

### 2.2. Orthogonal Basis Function Decomposition and Magnetic Anomaly Signal-Family Construction

Assume that the magnetic sensor moves along the X-axis. Its measurement trajectory can be expressed as $r(x)=[x,y_{0},z_{0}{]}^{T}$, where $y_{0}$ and $z_{0}$ denote the transverse offsets of the sensor trajectory relative to the magnetic dipole. To obtain a scale-independent representation of the magnetic anomaly signal, a dimensionless variable is introduced as u=x/d, where $d=\sqrt{y_{0}^{2}+z_{0}^{2}}$ is the closest distance from the measurement trajectory to the magnetic dipole. Substituting *u* into Equation (2) yields the magnetic anomaly vector along the measurement trajectory:

(3) $$\begin{array}{l} \Delta B=B_{r}=\frac{\mu_{0}}{4\pi d^{5}{\left(u^{2}+1\right)}^{5/2}}\left[\begin{array}{c} (2u^{2}-1)d^{2}m_{x}+3udy_{0}m_{y}+3udz_{0}m_{z}\\ (2y_{0}^{2}-u^{2}d^{2}-z_{0}^{2})m_{y}+3udy_{0}m_{x}+3y_{0}z_{0}m_{z}\\ (2z_{0}^{2}-u^{2}d^{2}-y_{0}^{2})m_{z}+3udz_{0}m_{x}+3y_{0}z_{0}m_{y} \end{array}\right]\\ =\frac{\mu_{0}}{4\pi d^{5}{\left(u^{2}+1\right)}^{5/2}}\left[\begin{array}{c} 2d^{2}m_{x}u^{2}+(3dy_{0}m_{y}+3dz_{0}m_{z})u-d^{2}m_{x}\\ -d^{2}m_{y}u^{2}+3dy_{0}m_{x}u+(2y_{0}^{2}m_{y}-z_{0}^{2}m_{y}+3y_{0}z_{0}m_{z})\\ -d^{2}m_{z}u^{2}+3dz_{0}m_{x}u+(2z_{0}^{2}m_{z}-y_{0}^{2}m_{z}+3y_{0}z_{0}m_{y}) \end{array}\right]\;\\ =\;\frac{\mu_{0}}{4\pi d^{5}{\left(u^{2}+1\right)}^{5/2}}\left[\begin{array}{ccc} 2d^{2}m_{x} & 3dy_{0}m_{y}+3dz_{0}m_{z} & -d^{2}m_{x}\\ -d^{2}m_{y} & 3dy_{0}m_{x} & 2y_{0}^{2}m_{y}-z_{0}^{2}m_{y}+3y_{0}z_{0}m_{z}\\ -d^{2}m_{z} & 3dz_{0}m_{x} & 2z_{0}^{2}m_{z}-y_{0}^{2}m_{z}+3y_{0}z_{0}m_{y} \end{array}\right]\left[\begin{array}{c} u^{2}\\ u\\ 1 \end{array}\right]\\ =\left[\begin{array}{ccc} a_{11} & a_{12} & a_{13}\\ a_{21} & a_{22} & a_{23}\\ a_{31} & a_{32} & a_{33} \end{array}\right]\left[\begin{array}{c} u^{2}/{\left(u^{2}+1\right)}^{5/2}\\ u/{\left(u^{2}+1\right)}^{5/2}\\ 1/{\left(u^{2}+1\right)}^{5/2} \end{array}\right] \end{array}$$

where $A=[a_{ij}]$ is a coefficient matrix, and its elements $a_{ij}$ are determined by the observation geometry, the orientation of the sensor coordinate system, and the direction of the target magnetic moment. It can be seen from Equation (3) that the vector magnetic anomaly signal measured along the trajectory can be linearly represented by three basis functions of *u*, namely,

(4) $$\phi_{1}(u)=u^{2}/(u^{2}+1{)}^{5/2},\phi_{2}(u)=u/(u^{2}+1{)}^{5/2},\phi_{3}(u)=1/(u^{2}+1{)}^{5/2}$$

To verify the linear independence of these basis functions, the Wronskian determinant is employed. The third-order Wronskian is defined as

(5) $$W(\phi_{1},\phi_{2},\phi_{3})(u)=\left|\begin{array}{ccc} \phi_{1} & \phi_{2} & \phi_{3}\\ \phi_{1}^{\prime } & \phi_{2}^{\prime } & \phi_{3}^{\prime }\\ \phi_{1}^{\prime \prime } & \phi_{2}^{\prime \prime } & \phi_{3}^{\prime \prime } \end{array}\right|$$

The calculation gives

(6) $$\begin{array}{l} W(u)=\frac{1}{{\left(u^{2}+1\right)}^{21/2}}\left|\begin{array}{ccc} u^{2} & u & 1\\ 2u-3u^{3} & 1-4u^{2} & -5u\\ 12u^{4}-21u^{2}+2 & 5u(4u^{2}-3) & 5(6u^{2}-1) \end{array}\right|\\ =\frac{1}{{\left(u^{2}+1\right)}^{21/2}}\cdot 2{\left(u^{2}+1\right)}^{3}=\frac{2}{{\left(u^{2}+1\right)}^{15/2}} \end{array}$$

Since $(u^{2}+1{)}^{15/2}>0$ for any u∈R, it follows that W(u)≠0. Therefore, the three basis functions in Equation (4) are linearly independent over the real domain.

The Gram–Schmidt procedure is then applied to the basis functions in Equation (4), yielding a set of orthogonal functions:

(7) $$\psi_{1}(u)=\phi_{1}(u)=\frac{1}{{\left(u^{2}+1\right)}^{5/2}}\;\psi_{2}(u)=\phi_{2}(u)-\frac{\langle \phi_{2},\psi_{1}\rangle }{\langle \psi_{1},\psi_{1}\rangle }=\frac{u}{{\left(u^{2}+1\right)}^{5/2}}\;\psi_{3}(u)=\phi_{3}(u)-\frac{\langle \phi_{3},\psi_{1}\rangle }{\langle \psi_{1},\psi_{1}\rangle }\psi_{1}-\frac{\langle \phi_{3},\psi_{2}\rangle }{\langle \psi_{2},\psi_{2}\rangle }\psi_{2}=\frac{u^{2}-1/7}{{\left(u^{2}+1\right)}^{5/2}}$$

The resulting orthogonal functions are further normalized in terms of energy:

(8) $$e_{i}(u)=\frac{\psi_{i}(u)}{\sqrt{\langle \psi_{i},\psi_{i}\rangle }},\;i=1,2,3$$

Thus, the normalized orthogonal basis functions are obtained as

(9) $$e_{1}(u)=\sqrt{\frac{128}{35\pi }}\frac{1}{{\left(1+u^{2}\right)}^{5/2}}\;e_{2}(u)=\sqrt{\frac{128}{5\pi }}\frac{u}{{\left(1+u^{2}\right)}^{5/2}}\;e_{3}(u)=\sqrt{\frac{56}{\pi }}\frac{u^{2}-1/7}{{\left(1+u^{2}\right)}^{5/2}}$$

To construct magnetic anomaly signals with adjustable amplitude, duration, and occurrence time, the above dimensionless orthogonal basis functions are extended into parameterized forms:

(10) $$f_{1}(t)=\sqrt{A\frac{128}{35\pi }}\cdot \frac{1}{{\left({\left(\frac{t-t_{0}}{w}\right)}^{2}+1\right)}^{5/2}}\;f_{2}(t)=\sqrt{A\frac{128}{5\pi }}\cdot \frac{\left(\frac{t-t_{0}}{w}\right)}{{\left({\left(\frac{t-t_{0}}{w}\right)}^{2}+1\right)}^{5/2}}\;f_{3}(t)=\sqrt{A\frac{56}{\pi }}\cdot \frac{{\left(\frac{t-t_{0}}{w}\right)}^{2}-\frac{1}{7}}{{\left({\left(\frac{t-t_{0}}{w}\right)}^{2}+1\right)}^{5/2}}$$

where A is the signal amplitude parameter, w is the signal width parameter, and $t_{0}$ is the temporal location parameter of the signal. This parameterized form enables the basis functions to represent magnetic anomaly signals with different amplitudes, durations, and occurrence times.

**Figure 2:**
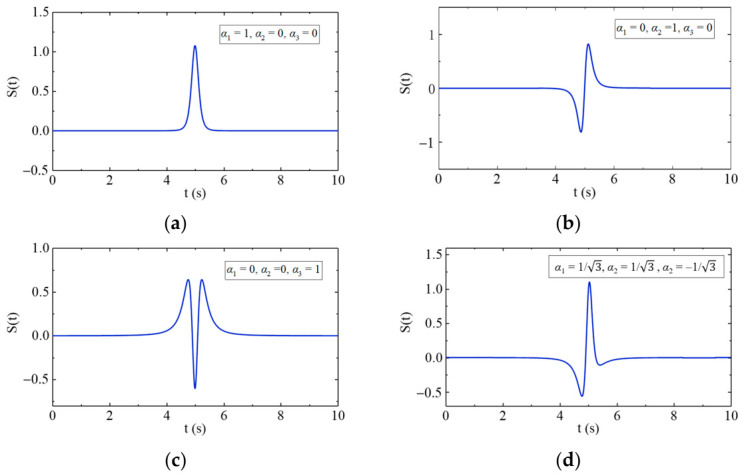
Representative signals in the magnetic anomaly signal family: (**a**) basis function *f*_1_; (**b**) basis function *f*_2_; (**c**) basis function *f*_3_; (**d**) a mixed-coefficient signal.

Therefore, under the magnetic dipole approximation, the magnetic anomaly signal along the measurement trajectory can be represented as a linear combination of the above orthogonal basis functions. The signal family formed by different combination coefficients is used to describe possible magnetic anomaly waveforms generated under different magnetic moment directions and encounter geometries. Accordingly, the vector magnetic anomaly signal family can be expressed as

(11) $$S=\left\{s\left(t\right)=\sum_{i=1}^{3}\alpha_{i}f_{i}\left(t\right)\left|\alpha_{1}^{2}+\alpha_{2}^{2}+\alpha_{3}^{2}=1\right.\right\}$$

where the combination coefficients $\alpha_{1},\alpha_{2},\alpha_{3}$ are associated with the target magnetic moment direction, observation geometry, and encounter angle. To eliminate amplitude-scale differences, the combination coefficients are energy-normalized. When A=1, w=0.15, and $t_{0}=5$, several representative signals sampled from the unit coefficient sphere are shown in Figure 2.

### 2.3. Biased Monostable Stochastic Resonance System

Stochastic resonance can be described as the forced stochastic motion of a particle in a one-dimensional overdamped system. The dynamical state of the particle is commonly governed by the Langevin equation:

(12) $$\frac{dx}{dt}=-\frac{dV(x)}{dx}+u(t)+\xi (t)$$

where x(t) denotes the response of the stochastic resonance system, V(x) is the nonlinear potential function, u(t) is the input excitation signal, and ξ(t) represents the noise term. This equation describes the trajectory of a Brownian particle moving in a nonlinear potential well under the joint action of the input signal and noise.

A typical potential function of an asymmetric monostable stochastic resonance system can be written as

(13) $$V(x)=-ax+\frac{b}{4}x^{4},\;b>0$$

where a and b are potential-function parameters. The condition b>0 ensures the monostable confinement of the potential function, allowing the particle to maintain a stable response within a finite potential-well region. Parameter a introduces an asymmetric bias into the potential function, thereby changing the bias direction and equilibrium position of the potential well. This potential function has only one stable equilibrium point, which is given by $x_{min}=\sqrt[{3}]{a/b}$.

Under the graphical convention used in this study, a<0 corresponds to a left-biased monostable potential function, whereas a>0 corresponds to a right-biased monostable potential function. Owing to the asymmetry of the potential function, a biased monostable stochastic resonance system is usually more sensitive to anomaly signals with a specific polarity [22].

**Figure 3:**
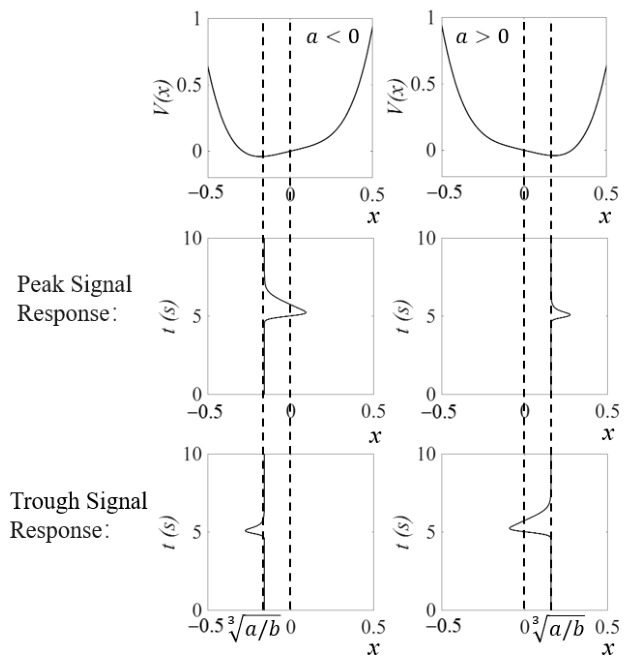
Responses of biased monostable stochastic resonance systems with different bias directions to the input signal.

As shown in Figure 3, under the same input signal, monostable stochastic resonance systems with different bias directions produce markedly different output responses. Specifically, the left-biased monostable potential is more favorable for enhancing peak-type anomaly signals, whereas its mirror-biased counterpart is more suitable for enhancing valley-type anomaly signals. This characteristic indicates that the biased monostable stochastic resonance system has clear polarity selectivity, providing a theoretical basis for the subsequent construction of the mirror dual-branch stochastic resonance detection framework.

In the simulations shown in Figure 3, the initial state of each biased monostable SR system was set to the stable minimum of the corresponding potential function. For the conventional biased monostable potential function, the initial condition was set as $x_{initial}=x_{min}=\sqrt[{3}]{a/b}$. This setting ensures that the particle starts from the static equilibrium state of the corresponding potential well.

## 3. Proposed Method

### 3.1. Multi-Parameter-Constrained Biased Monostable SR Model

A conventional biased monostable stochastic resonance system usually forms an asymmetric potential well by introducing a linear bias term into a monostable potential function. However, such a model contains only a limited number of tunable parameters, making it difficult to simultaneously regulate the potential-well position, local curvature, and nonlinear wall morphology. To improve the adaptability of the biased monostable SR system to non-periodic magnetic anomaly signals with varying morphologies, this study constructs a multi-parameter-constrained biased monostable potential function. By introducing a nonlinear odd-order term, the proposed potential function provides enhanced controllability over the potential-well shape.

The multi-parameter-constrained biased monostable potential function is defined as

(14) $$V\left(x\right)=-ax-\frac{b}{3}x^{3}+\frac{c}{4}x^{4},\left(a>0,b>0,c>0\;or\;a<0,b<0,c>0\right)$$

where a, b, and c are potential-shape control parameters. Parameter a mainly affects the linear bias of the potential function, b modifies the nonlinear skewness of the potential wall, and c constrains the high-order growth term. It should be noted that the effects of these three parameters are not independent. Instead, they jointly determine the location of the stable minimum, local curvature, and effective potential difference of the potential well. Compared with the conventional biased monostable potential function containing only a linear bias term, the introduced cubic term can modify the nonlinear growth characteristics of the potential walls on both sides, thereby providing a more flexible parameter space for dynamically enhancing non-periodic transient signals.

Based on the above potential function, the system dynamics under the joint action of the input signal and noise can be described by the following nonlinear Langevin equation:

(15) $$\frac{dx}{dt}=-\frac{dV(x)}{dx}+u(t)+\xi (t)=a+bx^{2}-cx^{3}+u(t)+\xi (t)$$

where x(t) denotes the output of the SR system, u(t) is the deterministic signal component in the noisy magnetic anomaly input, and ξ(t) represents the noise term.

Under multi-parameter constraints, the stochastic resonance mechanism of a one-dimensional biased monostable system can be interpreted as a dynamical enhancement process in which the particle undergoes intensified fluctuations around the stable point under the joint action of noise and the input signal and, under appropriate conditions, moves across the inflection point of the potential function [23]. Under the prescribed parameter constraints, the potential function maintains a monostable structure.

To clarify the dynamical characteristics of the proposed potential function, we further analyze its stationary point and inflection points. For the potential function V(x), the first-order and second-order derivatives are $V^{\prime }(x)=-a-bx^{2}+{cx}^{3}$ and $V^{\prime \prime }(x)=-2bx+{3cx}^{2}$. The stationary point is determined by $V^{\prime }(x)=$ 0, namely, $-2bx+{3cx}^{2}=0$. By applying the transformation x=y+b/3c, this cubic equation can be reduced to a depressed cubic equation. Its Cardano discriminant is $∆=a\left(27ac^{2}+4b^{3}\right)/108c^{4}$.

Under the parameter constraints used in this study, i.e., (a>0, b>0, c>0) or (a<0, b<0, c>0), the above discriminant is positive. Therefore, the stationary equation has only one real root. Since c>0, the potential function satisfies V(x)→+∞ as |x|→∞. Consequently, this unique stationary point corresponds to the stable minimum of the potential function.

Its stable minimum $x_{min}$ can be expressed as

(16) $$\left\{\begin{array}{l} x_{\min}=\frac{b}{3c}+\sqrt[{3}]{\frac{a}{2c}+\frac{b^{3}}{27c^{3}}+\sqrt{\Delta }}+\sqrt[{3}]{\frac{a}{2c}+\frac{b^{3}}{27c^{3}}-\sqrt{\Delta }}\\ \Delta =\frac{a(27ac^{2}+4b^{3})}{108c^{4}} \end{array}\right.$$

The inflection points of the potential function are determined by $V^{\prime \prime }(x)=0$, namely,

(17) $$\left\{\begin{array}{l} x_{1}=0\\ x_{2}=\frac{2b}{3c} \end{array}\right.$$

These two points are true inflection points because $V^{\prime \prime \prime }(0)=-2b\neq 0$ and $V^{\prime \prime \prime }(2b/3c)=-2b\neq 0$. For a>0,b>0,c>0, the stable minimum satisfies $x_{min}>2b/3c>0$; for a<0,b<0,c>0, the stable minimum satisfies $x_{min}<2b/3c<0$. Thus, the two parameter cases correspond to two mirror-biased monostable potentials. The sign constraints on a and b (a·b>0), together with c (c>0), are imposed to ensure monostability and to construct mirror potential wells with opposite polarity selectivity. In contrast, parameter combinations outside this domain no longer guarantee a unique stable minimum and are therefore excluded from the parameter search space.

**Figure 4:**
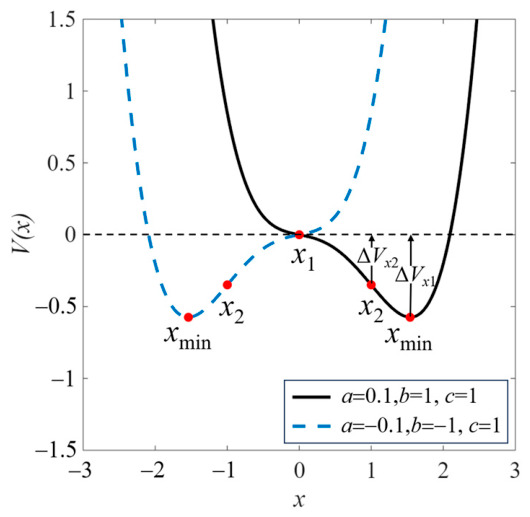
Potential-function model of the multi-parameter-constrained biased monostable system.

Figure 4 illustrates the multi-parameter-constrained biased monostable potential function, where the inflection points and the stable minimum are marked. Unlike bistable or multi-stable SR systems, a monostable SR system does not contain a conventional inter-well potential barrier. Instead, it produces a continuous dynamical response around a unique stable minimum. Therefore, this study does not use the barrier height to characterize the system response condition. Instead, the potential-energy difference between each inflection point and the stable minimum is defined as the effective potential difference, which characterizes the difficulty for the particle to deviate from its stable equilibrium position under the joint action of noise and the input signal. For each biased monostable potential function satisfying either (*a* > 0, *b* > 0, *c* > 0) or (*a* < 0, *b* < 0, *c* > 0), the effective potential differences between its inflection points and the corresponding stable minimum are expressed as follows:

(18) $$\left\{\begin{array}{l} \Delta V_{x1}=V(x_{1})-V(x_{\min})=\frac{3}{4}ax_{\min}+\frac{1}{12}bx_{\min}^{3}\\ \Delta V_{x2}=V(x_{2})-V(x_{\min})=-\frac{2ab}{3c}-\frac{4b^{4}}{81c^{3}}+\frac{3}{4}ax_{\min}+\frac{1}{12}bx_{\min}^{3} \end{array}\right.$$

This quantity characterizes the relative difficulty for the particle to deviate from the stable equilibrium region under the joint action of the input signal and noise. For the biased monostable potential considered here, the two inflection points are located on the same side of the stable minimum. When the particle moves away from the stable minimum along this potential-well wall, $x_{2}$ is the inflection point closer to the stable minimum, whereas $x_{1}$ is located farther away. These two inflection points divide the potential-well wall into distinct curvature regions. Therefore, ${∆V}_{x1}$ characterizes the potential-energy scale required for the particle to reach the first curvature-transition point near the stable equilibrium region, whereas ${∆V}_{x2}$ characterizes the potential-energy scale associated with a further deviation toward the curvature-transition point farther from the stable minimum. The two quantities are generally not identical because the biased monostable potential is asymmetric, and the two inflection points correspond to different positions, local slopes, and curvature-transition characteristics on the potential-well wall. This difference reflects the nonlinear wall morphology and the direction-dependent intrawell response of the biased monostable system.

When the system parameters are properly selected, the non-periodic signal, noise, and biased monostable system can interact synergistically. In this case, part of the noise energy is converted into a dynamical gain that is beneficial to the weak signal response, thereby enhancing the biased monostable stochastic resonance response and enabling the extraction of magnetic anomaly features under strong noise backgrounds.

**Figure 5:**
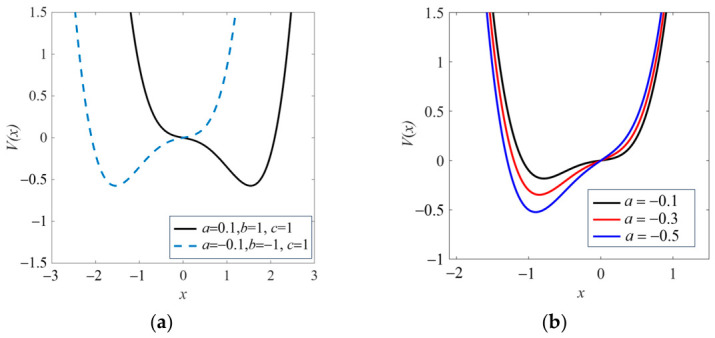

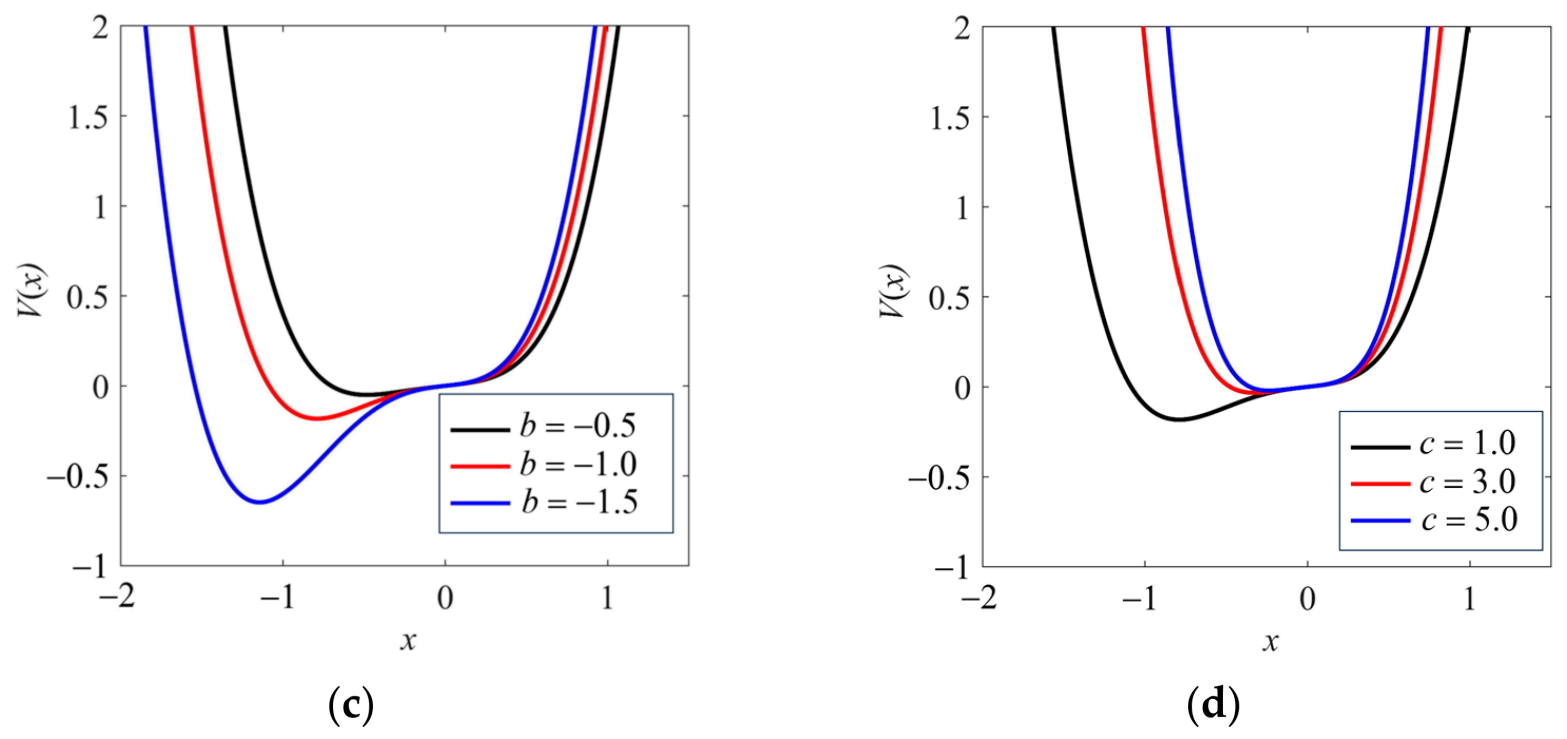
Biased monostable potential functions under different parameter settings: (**a**) Mirror-symmetric biased monostable potential functions with opposite bias directions; (**b**) variation with parameter *a*; (**c**) variation with parameter *b*; (**d**) variation with parameter *c*.

To intuitively illustrate the influence of each parameter on the potential shape, Figure 5 shows the potential-function curves under different parameter combinations. When the other parameters are fixed, increasing |a| mainly strengthens the bias of the potential function and changes the potential-energy difference near the stable point. Increasing |b| enhances the modulation effect of the cubic nonlinear term on the potential-wall morphology and increases the displacement of the inflection point $x_{2}$ relative to $x_{1}$. Increasing c strengthens the high-order confinement term, narrows the potential well, and modifies the effective potential difference. It should be emphasized that, in the actual system, the potential-well shape is jointly determined by a, b, and c; therefore, parameter optimization should be performed in the joint parameter space.

It should be noted that the conventional biased monostable system can already achieve polarity selection through the linear bias term. Therefore, the cubic term introduced in this study is not intended merely to realize polarity selection. Instead, it is introduced to increase the controllability of the potential-well morphology. Compared with the conventional potential function, the proposed multi-parameter-constrained potential function introduces a nonlinear bias force; this additional term modifies the nonlinear wall shape of the potential well and changes the locations of the inflection points and the effective potential difference. In particular, when the coefficient of the cubic term is set to zero, the proposed potential function reduces to the conventional linearly biased monostable potential function. Therefore, the conventional model can be regarded as a special case of the proposed model. The introduction of the cubic term provides an additional degree of freedom for adapting the SR dynamics to non-periodic magnetic anomaly signals with different morphologies.

The biased monostable stochastic resonance system in Equation (15) can be numerically solved using the fourth-order Runge–Kutta algorithm:

(19) $$\left\{\begin{array}{l} k_{1}=dt\left[a+bx{\left(n\right)}^{2}-cx{\left(n\right)}^{3}+s(n)\right]\\ k_{2}=dt\left[a+b{\left(x(n)+\frac{k_{1}}{2}\right)}^{2}-c{\left(x(n)+\frac{k_{1}}{2}\right)}^{3}+s(n)\right]\\ k_{3}=dt\left[a+b{\left(x(n)+\frac{k_{2}}{2}\right)}^{2}-c{\left(x(n)+\frac{k_{2}}{2}\right)}^{3}+s(n+1)\right]\\ k_{4}=dt\left[a+b{\left(x(n)+k_{3}\right)}^{2}-c{\left(x(n)+k_{3}\right)}^{3}+s(n+1)\right]\\ x(n+1)=x(n)+\frac{1}{6}(k_{1}+2k_{2}+2k_{3}+k_{4}) \end{array}\right.$$

where dt is the iteration step size, s(n) denotes the synthetic input signal composed of the magnetic anomaly signal u(n) and noise ξ(n), and x(n) is the output of the stochastic resonance system.

### 3.2. Mirror Dual-Branch SR Detection Framework

Because the biased monostable potential function has an asymmetric structure, both its stable equilibrium position and local potential-well morphology vary with the bias direction. Therefore, an SR system with a single bias direction is usually more sensitive to transient anomaly signals with a specific polarity [22,24]. Under different target magnetic moment directions and encounter geometries, magnetic anomaly signals may exhibit peak-type, valley-type, or bipolar waveforms. If only a single bias direction is used, the system may respond insufficiently to anomaly features with the opposite polarity. Therefore, parallel mirror branches are required to simultaneously enhance magnetic anomaly features with different polarities.

In this study, a multi-parameter-constrained mirror dual-branch biased monostable stochastic resonance system, denoted as MPC-MDBSR, is constructed. The system consists of two mirror-biased monostable SR branches, and their potential functions can be uniformly expressed as

(20) $$V_{\eta }\left(x\right)=-\eta ax-\eta \frac{b}{3}x^{3}+\frac{c}{4}x^{4},\eta \in \{+1,-1\}$$

where η=+1 and η=−1 denote the two mirror branches, respectively, and both branches share the same parameter set θ=(a,b,c). Let the outputs of the two branches be $x_{+}(n)$ and $x_{-}(n)$, respectively. Because the two branches may differ in static bias, output amplitude scale, and background fluctuation level, direct fusion may cause one branch to dominate numerically. Therefore, branch-wise baseline standardization is performed:

(21) $$g_{+}(n)=\frac{x_{+}(n)-\frac{1}{\left|X_{B}\right|}\sum_{n\in X_{B}}x_{+}(n)}{\sqrt{\frac{1}{\left|X_{B}\right|-1}\sum_{n\in X_{B}}{\left(x_{+}(n)-\frac{1}{\left|X_{B}\right|}\sum_{n\in X_{B}}x_{+}(n)\right)}^{2}}}$$

(22) $$g_{-}(n)=\frac{x_{-}(n)-\frac{1}{\left|X_{B}\right|}\sum_{n\in X_{B}}x_{-}(n)}{\sqrt{\frac{1}{\left|X_{B}\right|-1}\sum_{n\in X_{B}}{\left(x_{-}(n)-\frac{1}{\left|X_{B}\right|}\sum_{n\in X_{B}}x_{-}(n)\right)}^{2}}}$$

where $g_{+}(n)$ and $g_{-}(n)$ denote the normalized deviations of the two branch outputs relative to their respective background fluctuation scales. $X_{B}$ is the baseline interval. This operation makes each branch output referenced to its own background fluctuation scale, thereby reducing the influence of static-bias differences and amplitude-scale differences on subsequent fusion.

After baseline standardization, the two standardized branch outputs are regarded as a two-dimensional response vector, $g(n)=[g_{+}(n),g_{-}(n){]}^{T}$, and its Euclidean norm is defined as the joint response intensity of the dual-branch system:

(23) $$A(n)=\sqrt{g_{+}^{2}(n)+g_{-}^{2}(n)}$$

This intensity term describes the overall deviation of the dual-branch output from the baseline background at the current time. Since the responses of the two branches are combined in a squared form, A(n) avoids mutual cancelation between responses with different polarities during direct summation and is therefore suitable for characterizing target-presence intensity.

The intensity term A(n) can indicate whether an anomaly response is significant, but it cannot distinguish the directional attributes of peak-type, valley-type, or bipolar responses. To characterize the relative dominance of the two mirror branches, a normalized direction term is further defined as

(24) $$P(n)=\frac{g_{+}^{2}(n)-g_{-}^{2}(n)}{g_{+}^{2}(n)+g_{-}^{2}(n)+\varepsilon }$$

where ε>0 is a regularization constant used to avoid an excessively small denominator in low-energy regions and to suppress direction misjudgment in noise-dominated regions. P(n)∈[−1,1], where its sign indicates the dominant branch direction and its absolute value represents the degree of directional dominance. When the two branch responses are comparable, P(n) approaches zero; when one branch is clearly dominant, |P(n)| approaches one. Therefore, the direction term P(n) not only characterizes the relative polarity dominance of the dual-branch response but also reduces the contribution of background fluctuations without stable directional dominance in the subsequent direction-aware fused output.

Finally, the direction-aware fused output is constructed as

(25) y(n)=A(n)P(n)

This fusion form combines unsigned response intensity with normalized directional dominance, allowing the output to retain both magnetic anomaly response intensity and branch-polarity information. More importantly, the fused output is not a direct summation of the two branch responses. When the input is dominated by background noise, the standardized responses of the two mirror branches usually do not exhibit a stable branch dominance. In this case, $g_{+}(n)$ and $g_{-}(n)$ are more likely to be comparable, and P(n) approaches zero, thereby suppressing background fluctuations without clear directional dominance. In contrast, when a magnetic anomaly signal with a definite polarity characteristic is present, one branch becomes dominant, P(n) increases, and the fused output produces a significant response. Therefore, the proposed fusion mechanism uses the response difference between the two branches to enhance polarity-consistent anomaly features while reducing the risk of noise enhancement caused by simple response superposition.

**Figure 6:**
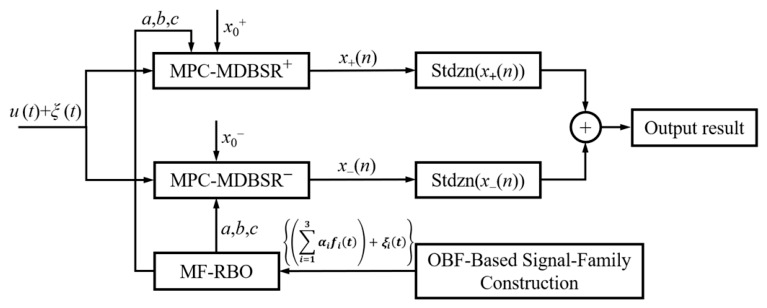
Schematic of the MPC-MDBSR detection framework.

As shown in Figure 6, the MPC-MDBSR system consists of two mirror-biased monostable SR systems, denoted as MPC-MDBSR+ and MPC-MDBSR−, respectively. MPC-MDBSR+ is mainly sensitive to peak-type anomaly components, while MPC-MDBSR− is mainly sensitive to valley-type anomaly components. The two biased monostable SR systems share the same parameter set θ=(a,b,c), and the final output is obtained through the direction-aware fusion mechanism. The complete detection procedure is as follows. First, the basis functions of vector magnetic anomaly signals are obtained through orthogonal basis function decomposition, and the magnetic anomaly signal family is constructed. Then, noisy synthetic training samples are generated from the signal family and used as inputs to the mirror dual-branch SR system for robust joint optimization. Next, a unified robust optimization objective is defined, and the multi-fidelity robust Bayesian optimization (MF-RBO) algorithm is employed to optimize the system parameters. The goal is to obtain a unified parameter set θ=(a,b,c) that provides good detection capability for different members of the signal family. Finally, the optimized parameters are used to drive the mirror dual-branch SR system for input-signal processing, and the final output is obtained through direction-aware fusion.

### 3.3. Composite Evaluation Metric Based on the Correlation Coefficient and Wavelet-Domain Image Structural Similarity Index

The waveform of a magnetic anomaly signal is jointly affected by the target magnetic moment direction, encounter velocity, encounter angle, and encounter distance. It usually appears as a short-duration, non-periodic, and morphologically variable transient signal. Therefore, its energy is not concentrated at a single fixed frequency but exhibits pronounced time-varying characteristics. A stochastic resonance system can be regarded as a nonlinear parameterized signal enhancer. Its output performance strongly depends on the selection of potential-function parameters, while the parameter-search process is directly guided by the evaluation metric.

However, conventional metrics do not always provide an effective assessment of magnetic anomaly recovery. For example, SNR mainly reflects the global energy ratio and cannot accurately indicate whether short-duration transient features are recovered. Kurtosis is sensitive to impulsive components but is also susceptible to spike noise. Although the correlation coefficient can effectively evaluate time-domain waveform similarity, it cannot adequately evaluate the time–frequency structure of the signal. Therefore, a composite metric that jointly accounts for time-domain waveform preservation and time–frequency structural recovery is required to guide the parameter optimization of the stochastic resonance system.

To analyze the sensitivity of different evaluation metrics to magnetic anomaly features, the wavelet-domain image peak signal-to-noise ratio (WD-PSNR) is first introduced. Specifically, the continuous wavelet transform is applied to both the reference signal u(t) and the SR output y(t), and their magnitude matrices are used as time–frequency representations. These magnitude matrices are then normalized to obtain the wavelet-domain images $I_{u}$ and $I_{y}$. WD-PSNR is defined as

(26) $$\mathrm{WD}\text{-}\mathrm{PSNR}=10\log_{10}\left(\frac{L^{2}}{\mathrm{MSE}(I_{u},I_{y})}\right)$$

where *L* denotes the maximum gray value of the normalized wavelet-domain image, and $MSE(I_{u},I_{y})$ is the mean squared error between the wavelet-domain images of the reference signal and the SR output.

The correlation coefficient (CC) is a commonly used time-domain similarity metric for evaluating non-periodic signal detection results. It measures the waveform consistency between the reference signal and the system output. Let u(n) denote the reference magnetic anomaly signal and y(n) denote the SR output.

(27) $$CC=\frac{\sum_{t=m}^{n}\left(u(t)-\bar{u}\right)\left(y(t)-\bar{y}\right)}{\sqrt{\sum_{t=m}^{n}{\left(u(t)-\bar{u}\right)}^{2}\sum_{t=m}^{n}{\left(y(t)-\bar{y}\right)}^{2}}}$$

where $\bar{u}$ and $\bar{y}$ are the mean values of the sequence u(t) and the sequence y(t), respectively.

To further evaluate the similarity of the SR output in terms of time–frequency structure, the wavelet-domain image structural similarity index (WD-SSIM) is adopted. Specifically, CWT is applied to both the reference signal u(t) and the SR output y(t), and the normalized CWT magnitude matrices are regarded as two-dimensional time–frequency images $I_{u}$ and $I_{y}$. Based on these images, WD-SSIM is defined as

(28) $$\mathrm{WD}\text{-}\mathrm{SSIM}\left(I_{u},I_{y}\right)=\frac{\left(2\mu_{u}\mu_{y}+C_{1}\right)}{\left(\mu_{u}^{2}+\mu_{y}^{2}+C_{1}\right)}\times \frac{\left(2\sigma_{u}\sigma_{y}+C_{2}\right)}{\left(\sigma_{u}^{2}+\sigma_{y}^{2}+C_{2}\right)}\times \frac{\left(\sigma_{uy}+C_{3}\right)}{\left(\sigma_{u}\sigma_{y}+C_{3}\right)}$$

where $\mu_{u}$ and $\mu_{y}$ are the mean values of the wavelet-domain images $I_{u}$ and $I_{y}$, respectively; $\sigma_{u}^{2}$ and $\sigma_{y}^{2}$ are their variances; $\sigma_{uy}$ is their covariance; and *C*_1_, *C*_2_, and *C*_3_ are stabilizing constants used to avoid excessively small denominators. A larger WD-SSIM indicates higher similarity between the SR output and the reference signal in terms of wavelet-domain time–frequency structure.

To compare the responses of different evaluation metrics to the time-domain morphology, occurrence time, and noise level of magnetic anomaly signals, the basis function *f*_1_ is used as the reference signal. The sensitivities of WD-PSNR, WD-SSIM, and CC to the coefficient-space deviation angle $\theta_{k}$, signal occurrence time $t_{0}$, and input SNR are then investigated. The combination coefficients in the signal family are parameterized using spherical coordinates:

(29) $$\left\{\begin{array}{l} \alpha_{1}=\cos\theta \\ \alpha_{2}=\sin\theta \cos\phi \\ \alpha_{3}=\sin\theta \sin\phi \\ \theta \in \left[0,\pi /2\right]\\ \phi \in \left[-\pi ,\pi \right) \end{array}\right.$$

Let the reference coefficient vector be $\alpha_{ref}={\left(1,0,0\right)}^{T}$ and the tested coefficient vector be $\alpha ={\left(\alpha_{1},\alpha_{2},\;\alpha_{3}\right)}^{T}$, and ‖α‖=1. The coefficient-space deviation angle $\theta_{k}$ is defined as

(30) $$\theta_{k}=\arccos\left(\alpha_{ref}^{T}\alpha \right)$$

where $\alpha_{ref}$ is the reference coefficient vector and α is the tested coefficient vector. The coefficient-space deviation angle $\theta_{k}$ quantifies the deviation of α from $\alpha_{ref}$ on the coefficient hemisphere. A larger $\theta_{k}$ indicates a stronger morphological deviation of the combined signal from the basis function *f*_1_.

**Figure 7:**
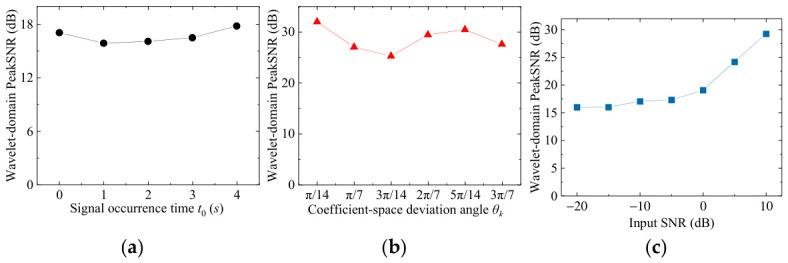

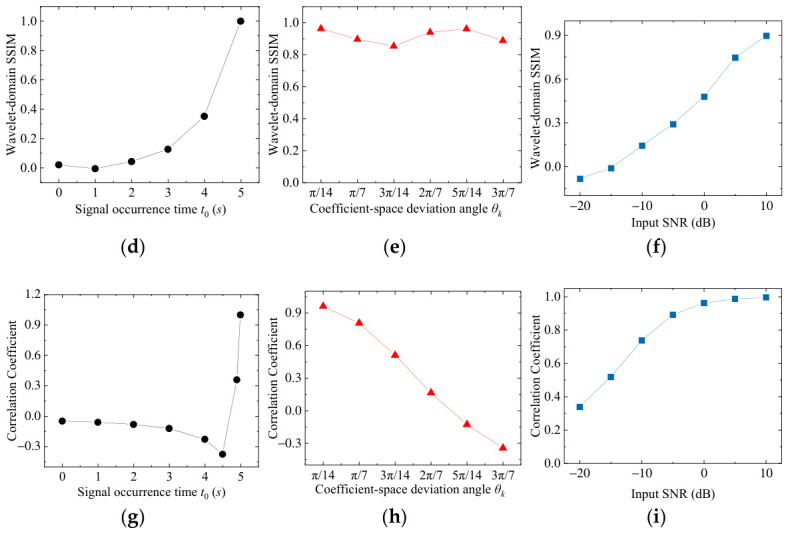
Sensitivity analysis of the evaluation metrics with respect to signal occurrence time, coefficient-space deviation angle, and input SNR: (**a**) variation in WD-PSNR with signal occurrence time; (**b**) variation in WD-PSNR with coefficient-space deviation angle; (**c**) variation in WD-PSNR with input SNR; (**d**) variation in WD-SSIM with signal occurrence time; (**e**) variation in WD-SSIM with coefficient-space deviation angle; (**f**) variation in WD-SSIM with input SNR; (**g**) variation in CC with signal occurrence time; (**h**) variation in CC with coefficient-space deviation angle; (**i**) variation in CC with input SNR.

Figure 7a,b show that WD-PSNR varies slowly when the signal occurrence time and coefficient-space deviation angle change. Figure 7c indicates that WD-PSNR changes only slightly when the input SNR ranges from −20 dB to −5 dB. When the input SNR ranges from −5 dB to 10 dB, WD-PSNR exhibits an approximately linear relationship with the input SNR. This suggests that WD-PSNR has limited sensitivity to signal morphology and occurrence time and is sensitive to noise-level variations only within a specific SNR range. Figure 7d–f show that WD-SSIM is sensitive to variations in signal occurrence time and input SNR and can reflect the recovery of wavelet-domain time–frequency structures. However, under the present test setting, its sensitivity to coefficient-space morphological variations is weaker than that of CC. Figure 7g–i show that CC is sensitive to signal morphology and input SNR, but its sensitivity to signal occurrence time is mainly evident in the local region near the true occurrence time.

Based on the results in Figure 7, WD-PSNR responds weakly to variations in signal occurrence time and coefficient-space deviation angle and is therefore unsuitable as a standalone parameter-optimization objective. WD-SSIM is sensitive to signal occurrence time and noise-level variations and can reflect the recovery of wavelet-domain time–frequency structures. However, under the current test setting, its sensitivity to time-domain waveform morphology is weaker than that of CC. In contrast, CC is more sensitive to time-domain waveform variations. Therefore, CC and WD-SSIM provide complementary information for evaluating the SR output: CC mainly measures local time-domain waveform consistency, whereas WD-SSIM measures the structural similarity of the normalized CWT magnitude images in the wavelet domain.

Since CC∈[−1,1], whereas WD-SSIM∈[0,1] in the present implementation, their value ranges are different and the sign of CC may affect the interpretation of the composite score. Therefore, CC is first linearly normalized as

(31) $${\mathrm{CC}}^{*}=\frac{\mathrm{CC}+1}{2}$$

Based on this normalization, the CC–WD-SSIM composite evaluation metric is constructed as

(32) $$S_{\mathrm{CC}\text{-}\mathrm{WDSSIM}}={\mathrm{CC}}^{*}\cdot \mathrm{WD}\text{-}\mathrm{SSIM}$$

The multiplicative form is used as a dimensionless composite optimization score. Its purpose is to impose a simultaneous constraint on time-domain waveform preservation and wavelet-domain time–frequency structural recovery. Compared with a linear additive form, the multiplicative form does not allow a high value of one sub-metric to fully compensate for a low value of the other. If a parameter set improves the local waveform correlation but damages the wavelet-domain time–frequency structure, or improves the wavelet-domain structural similarity while failing to preserve the local waveform, the corresponding composite score will be suppressed. Therefore, the proposed metric reduces the risk of parameter optimization being biased toward a single feature and provides a unified evaluation criterion for the subsequent robust Bayesian parameter optimization.

### 3.4. Multi-Fidelity Robust Bayesian Parameter Optimization

The detection performance of the MPC-MDBSR system is determined by the potential-function parameter vector θ=(a,b,c). However, there is no explicit analytical mapping from θ to the final detection performance, because each candidate parameter set must be evaluated through numerical SR simulations under different signal morphologies and noise realizations. Therefore, the parameter selection task is formulated as a noisy black-box optimization problem in a bounded continuous parameter space:

(33) $$\theta^{*}=\arg\underset{\theta \in \Theta }{\max}J(\theta )$$

where Θ denotes the parameter search space, and J(θ) is the robust evaluation objective constructed based on the magnetic anomaly signal family. Since each objective-function evaluation requires repeated executions of the stochastic resonance system under multiple signal morphologies and multiple noise realizations, direct high-fidelity global optimization would lead to a high computational cost. To address this issue, a multi-fidelity robust Bayesian optimization strategy is adopted to reduce the search cost while improving the robustness of parameter selection.

Assume that the evaluation signal family contains M representative signals $S=s_{i}\left(t\right),\left(i=1,\cdots ,M\right)$. For a given parameter vector θ, the CC-WD-SSIM composite score of the i-th signal after Monte Carlo noise realizations is denoted as $S_{CC-WDSSIMi}$, where $S_{CC-WDSSIMi}$ represents the statistical aggregation of repeated evaluations under different noise realizations. The robust objective is defined as

(34) $$J\left(\theta \right)=\frac{1}{M}\sum_{i=1}^{M}S_{\mathrm{CC}\text{-}\mathrm{WDSSIM}i}\left(\theta \right)-\lambda \mathrm{Var}\left(S_{\mathrm{CC}\text{-}\mathrm{WDSSIM}i}\left(\theta \right)\right)$$

The first term in Equation (34) represents the average enhancement performance of θ over the magnetic anomaly signal family, whereas the second term penalizes parameter combinations that produce large performance variations among different signal morphologies. The objective is not to obtain an optimal parameter set for a single magnetic anomaly waveform, but to determine a unified robust parameter set that provides stable detection capability for different members of the signal family.

To reduce the computational cost of robust optimization, a multi-fidelity optimization framework is constructed from two aspects: gradually increasing the complexity of the signal morphologies involved in the evaluation and progressively increasing the number of Monte Carlo repetitions. In this study, the fidelity level is increased from two aspects. The first aspect is signal-morphology fidelity. The low-fidelity stage uses only the three orthogonal basis functions as anchor signals, whereas the medium- and high-fidelity stages further include sparse mixed signals generated in the coefficient space. The second aspect is statistical fidelity. The number of Monte Carlo repetitions is gradually increased from MC0 to MC1 and MC2, so the evaluation becomes progressively less sensitive to individual noise realizations. Therefore, the optimization does not directly perform expensive high-fidelity evaluation over the full signal family at the beginning. Instead, it first identifies promising parameter regions using a simplified signal set and a small number of noise realizations, and then progressively verifies the robustness of candidate parameters using richer signal morphologies and more noise realizations. This design enables a balance between search efficiency and robust parameter selection.

Before the staged optimization, the sparse mixed-signal set is constructed from the three orthogonal basis functions. Specifically, the anchor signal set is defined as S0 = {*f*_1_, *f*_2_, *f*_3_}c, and the sparse mixed-signal set S1 is generated by sampling the coefficient space of $s(t)=\alpha_{1}f_{1}+\alpha_{2}f_{2}+\alpha_{3}f_{3}$, $\alpha_{1}^{2}+\alpha_{2}^{2}+\alpha_{3}^{2}=1$. The anchor signal set S0 represents three basic magnetic anomaly morphologies, whereas S1 contains mixed morphologies generated by different coefficient combinations. This hierarchical signal construction allows the optimizer to first search under representative basis signals and then verify whether the candidate parameters remain effective for combined signal shapes. In addition, logarithmic reparameterization is applied before Bayesian optimization because the parameters a, b, and c may differ substantially in magnitude. The transformed variables are defined as $u_{a}={log}_{10}a$, $u_{b}={log}_{10}b$, and $u_{c}={log}_{10}c$.

Bayesian optimization is then conducted in the transformed parameter space $u={\left[u_{a},u_{b},u_{c}\right]}^{T}$. After the optimization is completed, the physical parameters are recovered by $a=10^{u_{a}}$, $b=10^{u_{b}}$, and $c=10^{u_{c}}$. This transformation improves the search efficiency and avoids the inefficient exploration that may occur when parameters with different orders of magnitude are optimized directly in the original space. Specifically, the optimization process consists of four stages.

The first stage is a low-fidelity coarse search stage. In this stage, Bayesian optimization is performed only on the anchor signal set S0. For each candidate parameter θ proposed by the Bayesian optimizer, noisy input samples are generated by adding MC0 independent noise realizations to each signal in S0. The MPC-MDBSR system is then solved under this parameter, and the CC-WD-SSIM composite metric is calculated for each signal and noise realization. These metric values are aggregated to obtain a low-fidelity estimate of the robust objective. Because only three anchor signals and a relatively small number of Monte Carlo repetitions are used, this stage has low computational cost and is mainly used to identify potentially promising parameter regions. The output of this stage is an initial candidate set C1 rather than the final optimal parameter.

The second stage is a medium-fidelity robust search stage. This stage is initialized using the candidate set C1 obtained from the coarse search. The evaluation signal set is expanded from S0 to S0 ∪ S1 so that both the anchor basis signals and sparse mixed signals are included. For each candidate parameter, the system response is evaluated under MC1 noise realizations, and the robust objective is computed by considering both the average enhancement performance and the performance variation among different signal morphologies. This stage is used to test whether a parameter that performs well on the anchor signals can still maintain stable performance for mixed magnetic anomaly waveforms. Parameters that perform well only for a single basis signal but fail on mixed morphologies are therefore filtered out. The output of this stage is a finalist parameter set C2.

The third stage is a high-fidelity re-evaluation stage. Instead of continuing global exploration over the entire parameter space, this stage focuses on the finalist set C2 obtained in the second stage. Each finalist parameter is re-evaluated on S0 ∪ S1 using a larger number of Monte Carlo repetitions MC2. The purpose of this stage is to reduce the uncertainty caused by random noise realizations and to obtain a statistically more reliable estimate of the robust objective. After this high-fidelity re-evaluation, the parameter with the best robust objective is selected as the current best parameter $\theta^{*}$.

The fourth stage is a neighborhood validation stage. To examine whether the current best parameter is a stable local solution rather than an isolated result caused by random evaluation fluctuations, local neighboring points are sampled around $\theta^{*}$ in the transformed parameter space. These neighboring parameters are evaluated on S0 ∪ S1 using the same robust objective. If the neighboring points produce comparable objective values, the selected parameter is considered locally stable. If a neighboring parameter achieves a better and stable robust objective, $\theta^{*}$ is updated accordingly. This neighborhood validation step improves the reliability of the final parameter selection and reduces the risk of selecting a parameter located at a narrow or unstable optimum.

After the four stages, the optimized parameter vector is recovered from the logarithmic parameter space and used as the unified robust parameter set for the subsequent mirror dual-branch SR signal processing. The pseudo-code of the proposed algorithm is presented as follows.

**Figure 8:**
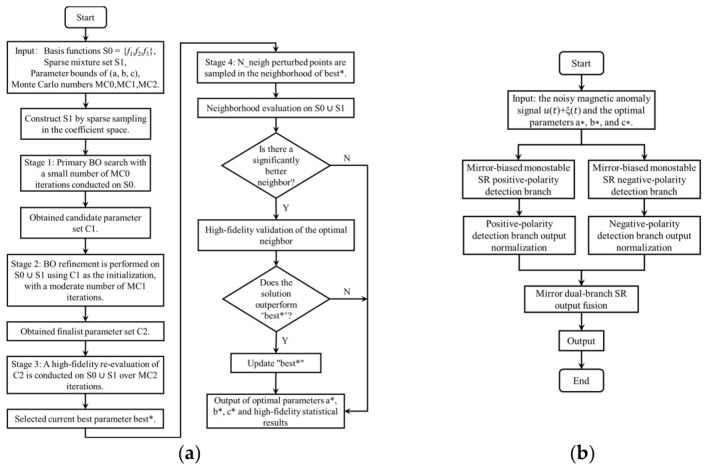
Workflow of multi-fidelity robust Bayesian optimization and signal processing: (**a**) parameter optimization; (**b**) signal processing using the mirror dual-branch SR system.

The proposed parameter optimization and signal processing procedure is illustrated in Figure 8. Figure 8a corresponds to the MF-RBO parameter optimization procedure described in Algorithm 1. First, the magnetic anomaly signal family is constructed from the orthogonal basis functions. The anchor signal set S0 consists of the three basis functions, whereas the sparse mixed-signal set S1 is generated by sampling the normalized coefficient space. Different noise realizations are then superimposed on these signals to form noisy evaluation samples. Second, the parameter vector θ=(a,b,c) is transformed into the logarithmic search space, and Bayesian optimization is performed in a staged manner. The low-fidelity stage searches promising regions using S0 and MC0 evaluations. The medium-fidelity stage expands the evaluation set to S0 ∪ S1 and uses MC1 evaluations to screen candidate parameters with better cross-morphology robustness. The high-fidelity stage re-evaluates the finalist set using MC2 evaluations to reduce the influence of random noise realizations. Finally, neighborhood validation is performed around the current best parameter to verify local stability. Through this procedure, a unified robust parameter set $\theta^{*}=(a^{*},b^{*},c^{*})$ is obtained for the magnetic anomaly signal family.
**Algorithm 1.** Multi-fidelity Robust Bayesian Optimization*Input: Basis functions S*0 *=* {*f*_1_*, f*_2_*, f*_3_}*, Sparse mixture set S*1*, Parameter bounds of (a, b, c), Robustness penalty coefficient λ, Monte Carlo numbers MC*0 *< MC*1 *< MC*2. *1: Construct S1 by sparse sampling in the coefficient space* *s = α*1*f1 *+ α*2*f2 *+ α*3*f3*, α*1^2^*+α*2^2^*+α*3^2^*=*1. *2: Apply logarithmic reparameterization:* *ua = log*10*(a), ub = log*10*(b), uc = log*10*(c).* *3: Stage* 1 *(coarse BO on S*0*):* *Perform Bayesian optimization on S*0 *using MC*0 *evaluations,* *and obtain a candidate set C*1. *4: Stage* 2 *(robust BO on S*0 *∪ S*1*):* *Initialize the second BO with C*1*,* *optimize the aggregated objective on S*0 *∪ S*1 *using MC*1 *evaluations,* *and obtain a finalist set C*2. *5: Stage* 3 *(high-fidelity review):* *Re-evaluate all finalists in C*2 *on S*0 *∪ S*1 *using MC*2 *evaluations,* and select the current best parameter θ*. *6: Stage* 4 *(neighborhood verification):* Sample local neighbors around θ*, *evaluate them on S*0 *∪ S*1*,* and update θ* if a better neighbor is confirmed. *7: Recover physical parameters:* *a =* 10*^ua^, b =* 10*^ub^, c =* 10*^uc^.* *Output:* Optimal parameters (a*, b*, c*) and corresponding robust objective.

Figure 8b shows the signal processing procedure after the robust parameters have been determined. For a given noisy magnetic anomaly input, the signal is simultaneously fed into two mirror-biased monostable SR branches with the optimized parameter set. The two branch outputs are then baseline-standardized to remove static bias and amplitude-scale differences. The standardized outputs are further used to construct the joint response intensity and the normalized direction term. Finally, the direction-aware fused output is obtained. Therefore, Figure 8a describes the offline robust parameter optimization process, whereas Figure 8b describes the online signal processing process using the optimized mirror dual-branch SR system.

## 4. Experimental Results and Analysis

### 4.1. Simulation Experiments with Colored Noise

To verify the detection capability of the proposed MPC-MDBSR method under colored-noise backgrounds, simulation experiments are first conducted using the vector magnetic anomaly signal family constructed in Section 2.2. Since real geomagnetic noise usually exhibits colored-noise characteristics, and its noise exponent α generally ranges from 0.5 to 1.5, $1/f^{\alpha }$ colored noise is adopted as the input noise model in the simulations [25] with α=0.7. The sampling frequency is set to $f_{s}=1000\;Hz$. Noisy input signals under different SNR conditions are generated by changing the amplitude of the magnetic anomaly signal. The SNR is defined as the ratio of signal power to noise power:

(35) $$\mathrm{SNR}=10\log_{10}\left(\frac{\sum_{i=1}^{N}u{\left(i\right)}^{2}}{\sum_{i=1}^{N}\xi {\left(i\right)}^{2}}\right)$$

where u(i) denotes the noise-free magnetic anomaly signal, ξ(i) denotes the colored noise, and *N* is the signal length.

**Figure 9:**
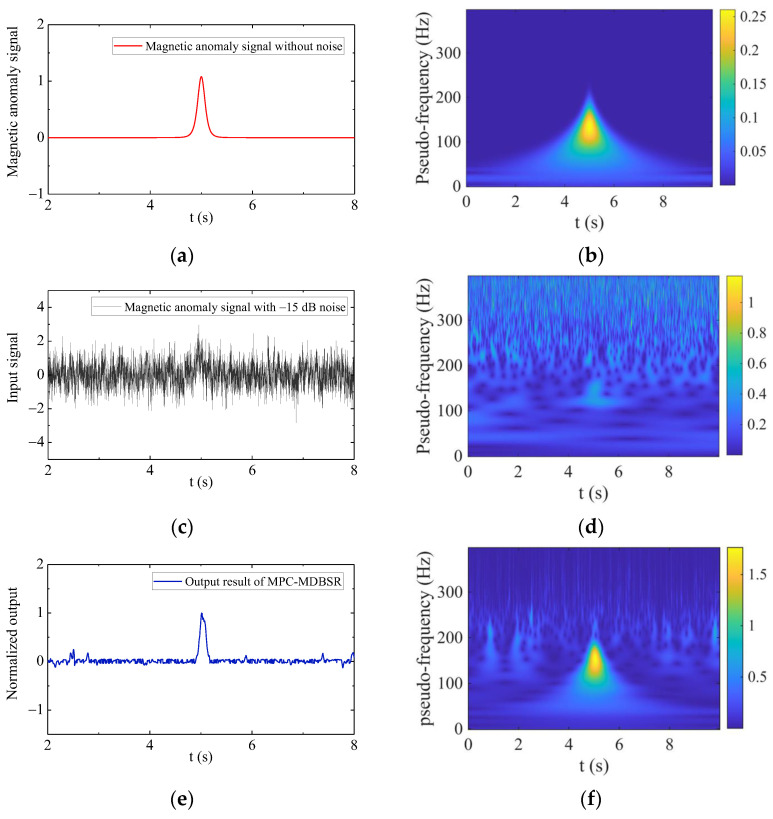
Detection results of the proposed method under −15 dB colored noise: (**a**) time-domain waveform of the noise-free magnetic anomaly signal; (**b**) CWT time–frequency representation of the noise-free magnetic anomaly signal; (**c**) time-domain waveform of the magnetic anomaly signal under −15 dB colored noise; (**d**) CWT time–frequency representation of the magnetic anomaly signal under −15 dB colored noise; (**e**) time-domain waveform of the output signal obtained by the proposed method; (**f**) CWT time–frequency representation of the output signal obtained by the proposed method.

Figure 9 shows the detection result of the proposed method for the basis function *f*_1_ under –15 dB colored noise. Figure 9a,b show the noise-free magnetic anomaly signal and its CWT time–frequency representation, respectively. Figure 9c,d show the noisy input signal contaminated by –15 dB colored noise and its CWT time–frequency representation, respectively. It can be seen that, under colored-noise interference, the original magnetic anomaly waveform is severely submerged in the time domain, and its local time–frequency structure is also significantly disturbed. After processing by MPC-MDBSR, the output signal in Figure 9e presents a clear response near the target occurrence time, and the CWT time–frequency structure in Figure 9f remains reasonably consistent with that of the noise-free reference signal. These results indicate that the proposed method can effectively detect the occurrence of magnetic anomaly signals under low-SNR colored-noise conditions and can recover their local time-domain features and time–frequency structural characteristics to a certain extent.

**Figure 10:**
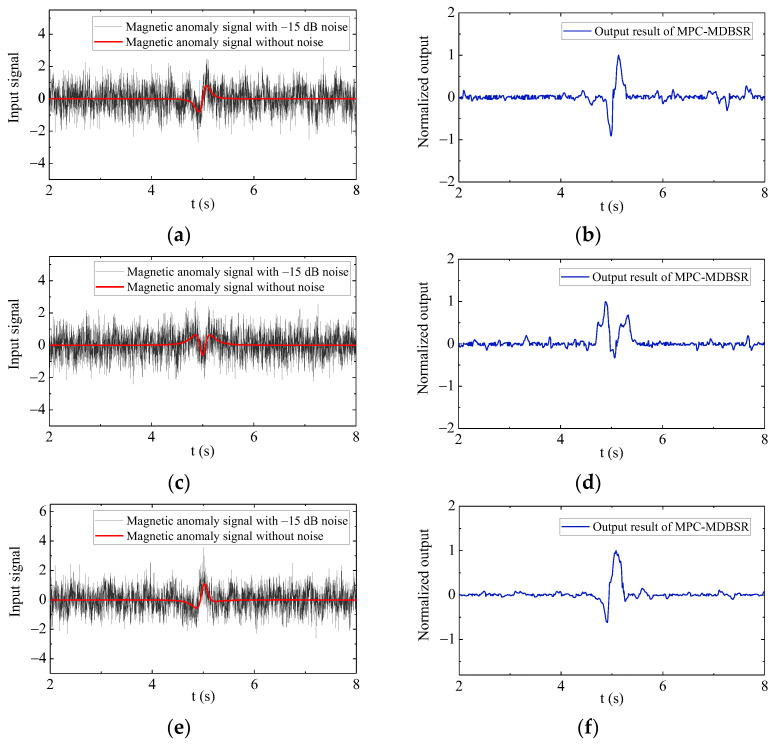
Detection results of the proposed method for magnetic anomaly signals with different morphologies: (**a**) basis function *f*_2_ signal with −15 dB colored noise; (**b**) time-domain detection result of the basis function *f*_2_ signal; (**c**) basis function *f*_3_ signal with −15 dB colored noise; (**d**) time-domain detection result of the basis function *f*_3_ signal; (**e**) mixed-coefficient signal with −15 dB colored noise; (**f**) time-domain detection result of the mixed-coefficient signal.

To further verify the adaptability of the proposed method to magnetic anomaly signals with different morphologies, −15 dB colored noise is superimposed on the basis function *f*_2_, the basis function *f*_3_, and a mixed-coefficient signal. Figure 10a,c,e show the three types of noisy input signals, while Figure 10b,d,f show the corresponding MPC-MDBSR outputs. The results show that, although different basis functions and mixed signals have different polarities, waveform symmetries, and local variation characteristics, the proposed method can still produce clear responses within the target time window and recover their main waveform structures to some extent. This demonstrates that, owing to the mirror dual-branch structure and unified robust parameter optimization, MPC-MDBSR has good adaptability to multiple typical morphologies in the magnetic anomaly signal family.

### 4.2. Experiments with Measured Geomagnetic Noise

**Figure 11:**
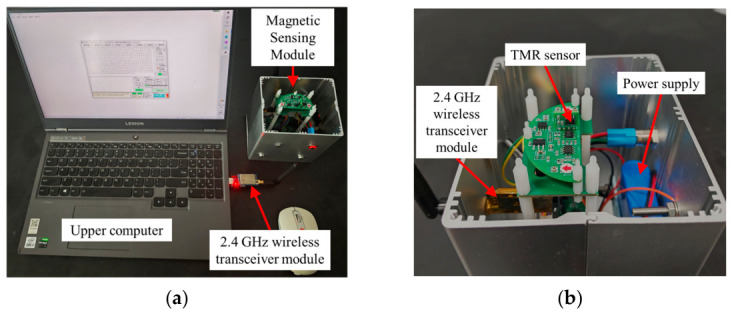
Magnetic-field measurement system: (**a**) overall system; (**b**) internal structure of the measurement module.

To verify the applicability of the proposed method under measured environmental noise, magnetic-field measurement experiments were conducted in an outdoor open area to acquire real geomagnetic noise data. The measurement system consisted of a tunnel magnetoresistance (TMR) sensor (TMR9082), a signal conditioning circuit, a signal acquisition and storage circuit, a wireless transceiver circuit, and a host computer for wireless data reception and readback. The sampling frequency of the signal acquisition and storage circuit was set to 1 kHz. The magnetic-field measurement system is shown in Figure 11.

The main specifications of the TMR-based magnetic measurement system used in this experiment are summarized in Table 1.

**Figure 12:**
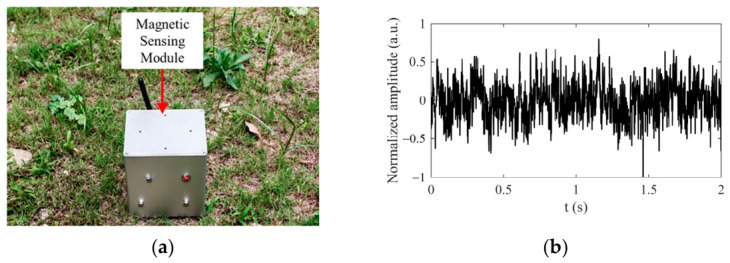

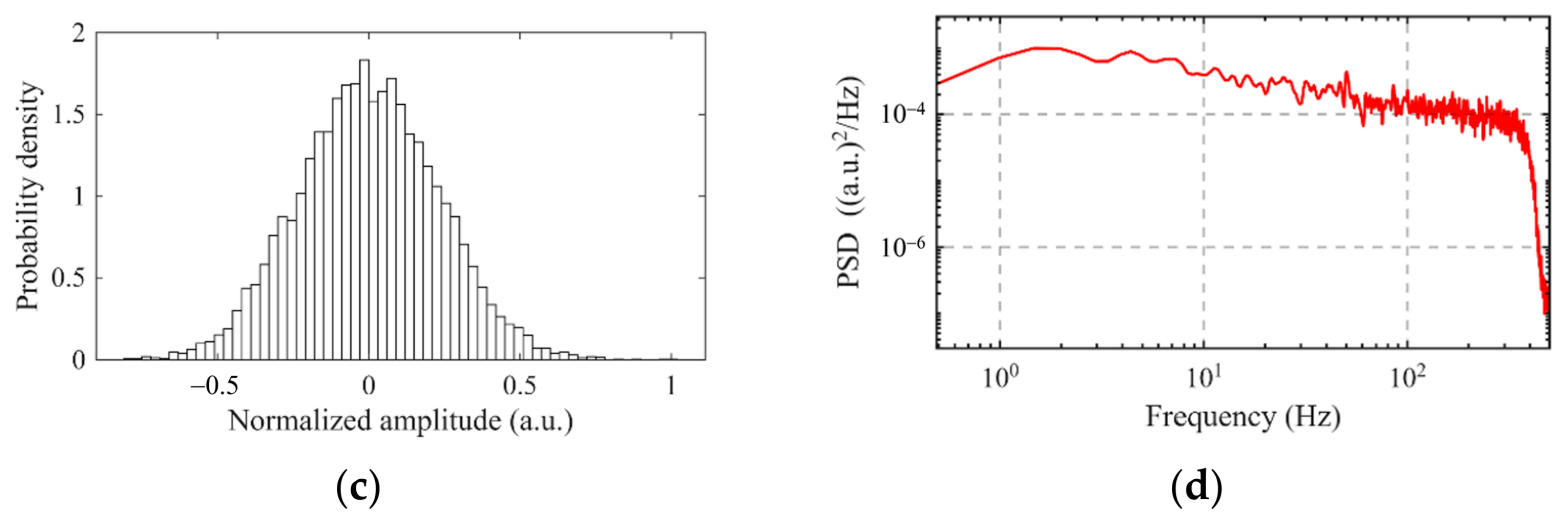
Characteristics of the measured geomagnetic noise: (**a**) measurement scene; (**b**) noise sample; (**c**) normalized histogram; (**d**) power spectral density.

Figure 12 presents the measured geomagnetic noise and its statistical characteristics. Figure 12a shows the noise acquisition scene, and Figure 12b shows a representative short-time segment of the noise sample acquired by the geomagnetic measurement device in an open outdoor environment. The noise sample contains not only background geomagnetic disturbances but also the intrinsic noise of the TMR sensor and the noise introduced by the sensor front-end analog circuit. Therefore, it better represents the noise environment in a practical measurement system. Figure 12c shows the normalized histogram of the measured data. Its probability distribution is approximately Gaussian, with a standard deviation of 0.229, skewness of 0.0104, and kurtosis of 3.111. Figure 12d shows the power spectral density of the noise sample. In the frequency range of 5~200 Hz, the noise power spectrum approximately follows a $1/f^{\alpha }$ decay with increasing frequency, with a noise exponent of α=0.646.

**Figure 13:**
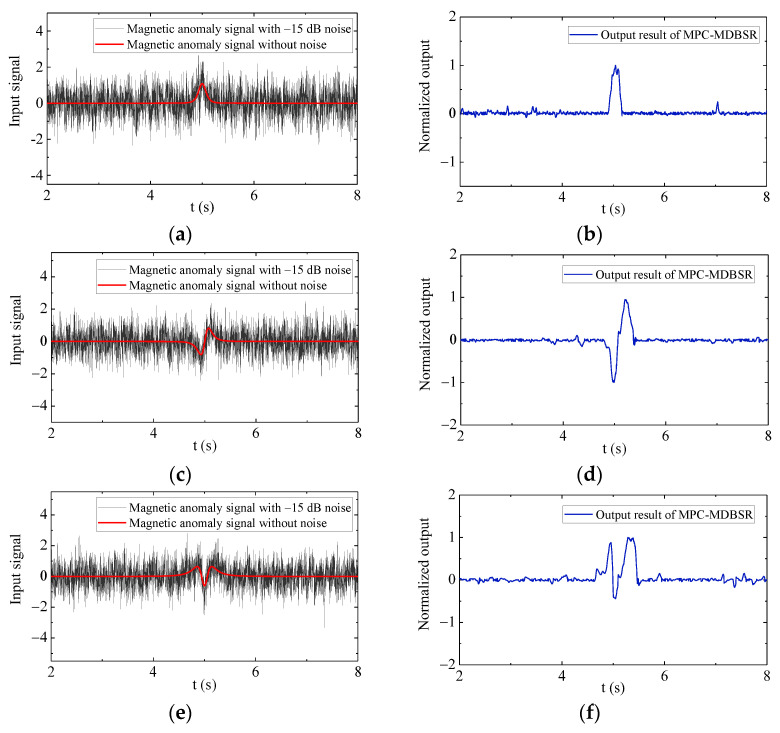

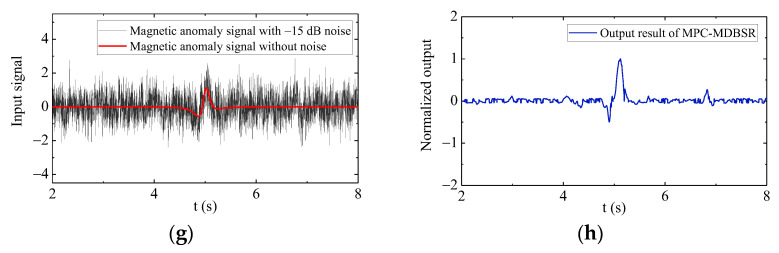
Detection results of the proposed method for magnetic anomaly signals with different morphologies under measured geomagnetic noise: (**a**) basis function f1 signal with −15 dB measured geomagnetic noise; (**b**) time-domain detection result of the basis function f1 signal; (**c**) basis function f2 signal with −15 dB measured geomagnetic noise; (**d**) time-domain detection result of the basis function f2 signal; (**e**) basis function f3 signal with −15 dB measured geomagnetic noise; (**f**) time-domain detection result of the basis function f3 signal; (**g**) mixed-coefficient signal with −15 dB measured geomagnetic noise; (**h**) time-domain detection result of the mixed-coefficient signal.

The measured noise sample was then superimposed on typical magnetic anomaly signals to evaluate the performance of the proposed method under real noise conditions. Figure 13 shows the detection results of the proposed method under measured geomagnetic noise. Figure 13a,c,e,g show the basis function *f*_1_, basis function *f*_2_, basis function *f*_3_, and mixed-coefficient signal contaminated by measured geomagnetic noise, respectively. The SNRs of all input synthetic signals were set to −15 dB. Figure 13b,d,f,h show the corresponding MPC-MDBSR outputs. It can be seen that, under measured geomagnetic noise interference, magnetic anomaly signals with different morphologies are clearly contaminated in the original inputs. Nevertheless, the proposed method still produces distinct responses near the target time window and preserves the main time-domain structures of the target signals. These results demonstrate that MPC-MDBSR is applicable not only to simulated colored-noise scenarios but also to measured noise conditions involving real geomagnetic background disturbances, sensor noise, and front-end circuit noise.

### 4.3. Performance Comparison with Existing Methods

**Figure 14:**
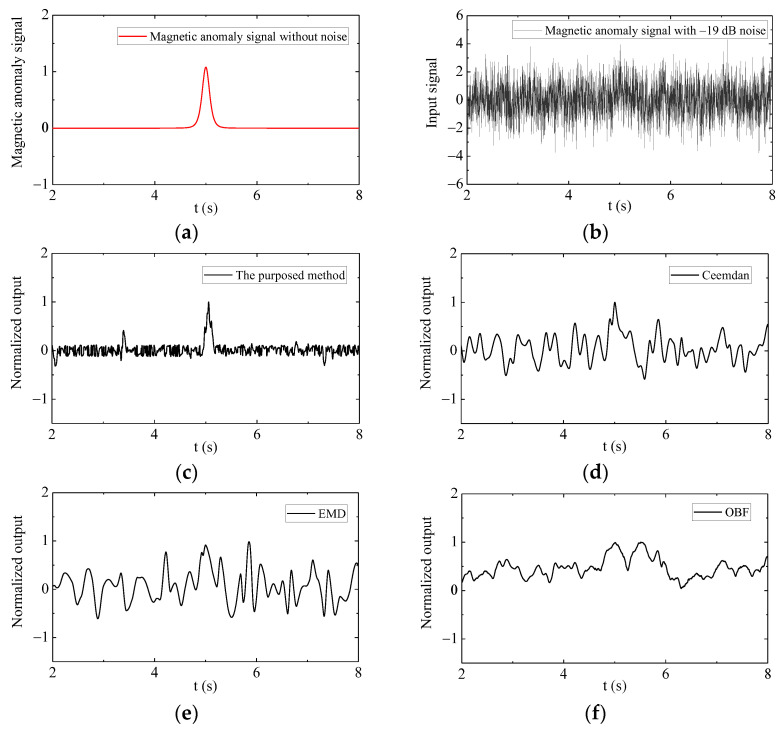
Comparison of detection results obtained by different methods under −19 dB measured geomagnetic noise: (**a**) noise-free magnetic anomaly signal; (**b**) noisy magnetic anomaly signal under −19 dB measured geomagnetic noise; (**c**) detection result of the proposed method; (**d**) detection result of CEEMDAN; (**e**) detection result of EMD; (**f**) detection result of OBF.

**Figure 15:**
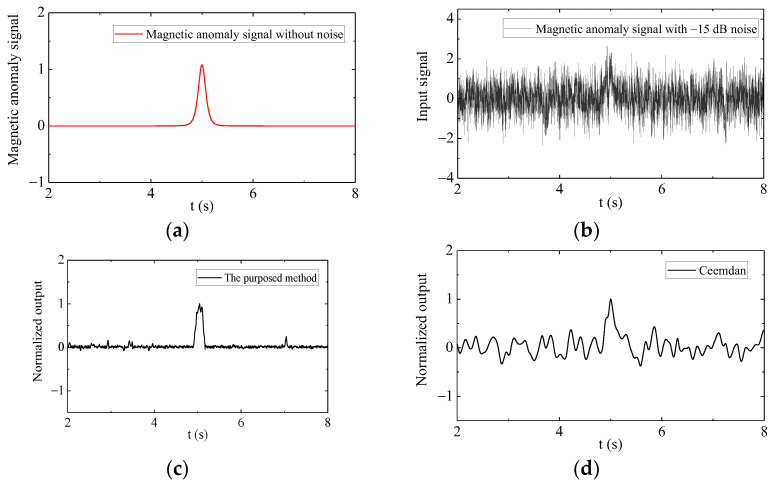

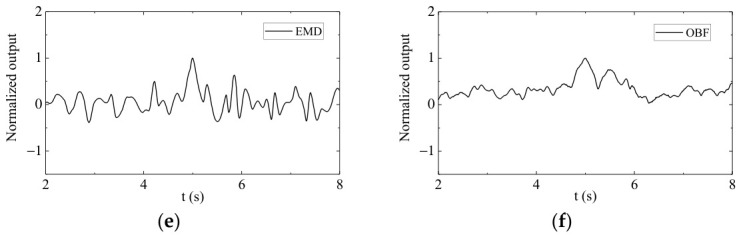
Comparison of detection results obtained by different methods under −15 dB measured geomagnetic noise: (**a**) noise-free magnetic anomaly signal; (**b**) noisy magnetic anomaly signal under −15 dB measured geomagnetic noise; (**c**) detection result of the proposed method; (**d**) detection result of CEEMDAN; (**e**) detection result of EMD; (**f**) detection result of OBF.

To further demonstrate the practical applicability of the proposed method in magnetic anomaly detection, three representative methods are selected for comparison with MPC-MDBSR: orthogonal basis function decomposition (OBF), empirical mode decomposition (EMD), and complete ensemble empirical mode decomposition with adaptive noise (CEEMDAN). To ensure a fair comparison, all methods are tested under the same input signals, SNR conditions, and evaluation metrics. To highlight the detection differences under low-SNR conditions, two input cases with measured geomagnetic noise at −19 dB and −15 dB are considered. The results are shown in Figure 14 and Figure 15, respectively.

Figure 14 compares the detection results obtained by different methods under −19 dB measured geomagnetic noise. Figure 14a shows the noise-free magnetic anomaly signal, and Figure 14b shows the noisy magnetic anomaly signal after adding −19 dB measured geomagnetic noise. It can be observed that, under this SNR condition, the time-domain waveform of the target signal is completely submerged by the measured noise, and the target peak structure and occurrence time corresponding to the noise-free reference signal can hardly be identified from the raw input. After MPC-MDBSR processing, Figure 14c exhibits a pronounced response near the target occurrence time, while the background noise is effectively suppressed and the local target feature becomes more prominent. In contrast, the target anomaly features in the outputs of CEEMDAN, EMD, and OBF are relatively weak and are difficult to distinguish reliably from the residual noise. Under the −15 dB condition, Figure 15c shows that MPC-MDBSR can better recover the main peak feature of the target signal. By comparison, although the other methods can reveal the target response to some extent, their residual noise remains evident, and their ability to preserve the local waveform structure is relatively limited. Overall, the proposed method exhibits better noise suppression and target-feature preservation under low-SNR measured geomagnetic noise.

**Figure 16:**
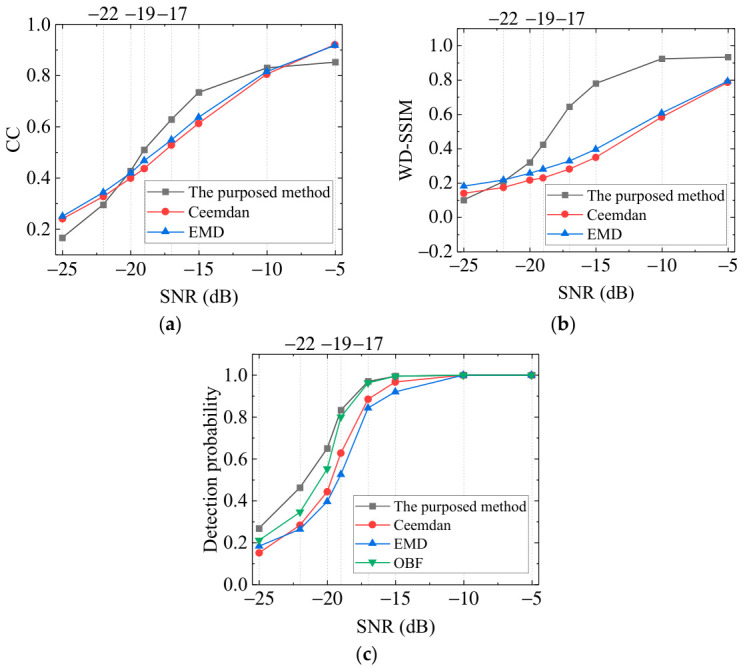
Comparison of detection performance among different methods: (**a**) correlation coefficient; (**b**) wavelet-domain image structural similarity index; (**c**) detection probability.

To further quantitatively compare the detection performance of different methods, Monte Carlo simulations are conducted to calculate the correlation coefficient, the wavelet-domain image structural similarity index, and the detection probability of magnetic anomaly signals under different SNR conditions. Figure 16 presents the statistical results obtained from 3000 repeated detection trials.

Figure 16a shows that, as the SNR increases, the correlation coefficients between the outputs of different methods and the reference signal generally increase. In the range from −20 dB to −10 dB, the correlation coefficient of MPC-MDBSR is higher than those of EMD and CEEMDAN, indicating better time-domain waveform preservation under strong noise. As the SNR further increases, the correlation coefficient of MPC-MDBSR gradually stabilizes at approximately 0.84, showing a saturation tendency. This phenomenon may be related to the nonlinear dynamical response of the SR system and the constraint of unified robust parameters. That is, the adopted parameter set is not locally optimized for a single SNR level or a single waveform but is obtained through robust optimization over the magnetic anomaly signal family and multiple noise conditions. Figure 16b shows that, when the SNR is higher than −22 dB, the WD-SSIM of MPC-MDBSR is generally higher than those of the comparison methods, indicating that the proposed method better preserves the time–frequency structure of magnetic anomaly signals in the wavelet domain. Figure 16c shows that, within the tested SNR range, the detection probability of the proposed method is generally higher than those of OBF, EMD, and CEEMDAN, demonstrating the more stable target-detection capability of MPC-MDBSR under low-SNR conditions.

The computational cost of the proposed method consists of two parts. The first part is the offline parameter optimization stage, in which multi-fidelity robust Bayesian optimization and Monte Carlo re-evaluation are performed over multiple signal morphologies and noise realizations. This stage is computationally expensive and requires several hours. However, this cost is incurred only before deployment to obtain a unified robust parameter set. Once the optimized parameter set is determined, Bayesian optimization does not need to be repeated during online detection. The second part is the online signal-processing stage. In this stage, the input signal is processed by two mirror-biased monostable SR branches, followed by baseline standardization, joint response-intensity calculation, direction-term calculation, and direction-aware fusion. Therefore, the online computation mainly consists of two one-dimensional numerical integrations and several algebraic operations. Its computational complexity increases approximately linearly with the signal length.

**Figure 17:**
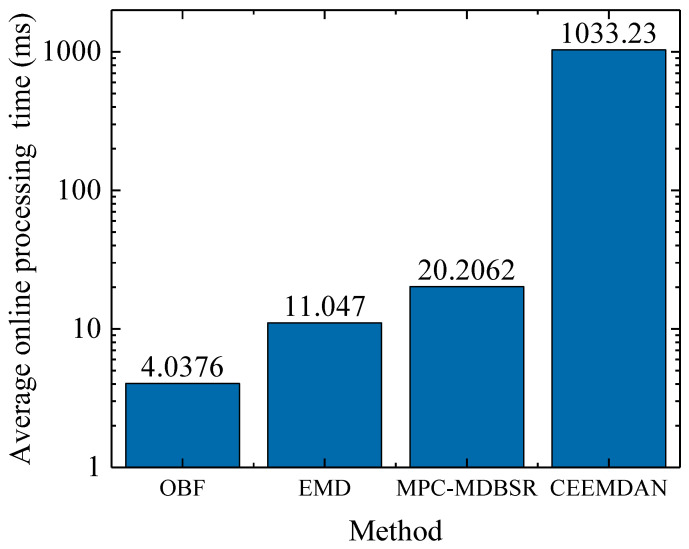
Comparison of the average running time of different methods.

As shown in Figure 17, the average online processing times of OBF, EMD, MPC-MDBSR, and CEEMDAN are 4.0376 ms, 11.047 ms, 20.2062 ms, and 1033.23 ms, respectively. The online runtime of MPC-MDBSR is higher than those of OBF and EMD, mainly because two nonlinear SR branches need to be solved and fused. However, it remains at the millisecond level under the tested condition and is much lower than that of CEEMDAN. Therefore, although the proposed framework is more complex than OBF and EMD, its online computational cost is still moderate, especially considering its improved low-SNR detection probability and better time–frequency structural preservation.

In summary, the experiments with simulated colored noise, measured geomagnetic noise, and comparative methods all show that MPC-MDBSR can effectively detect magnetic anomaly signals under low-SNR conditions. It also achieves favorable overall performance in terms of time-domain waveform preservation, time–frequency structural recovery, and detection probability.

## 5. Conclusions

This paper proposes a magnetic anomaly signal detection method based on a multi-parameter-constrained mirror dual-branch biased monostable stochastic resonance system for weak magnetic anomaly detection under complex noise backgrounds. First, a magnetic anomaly signal family is constructed through orthogonal basis function decomposition. Nonlinear odd-order bias terms are then introduced into the conventional biased monostable potential function to enhance the controllability of the potential-well width, depth, and wall morphology. A mirror dual-branch SR detection framework is further developed to improve the adaptability of the system to peak-type, valley-type, and bipolar magnetic anomaly signals. In addition, a CC–WD-SSIM composite evaluation metric is proposed to jointly constrain time-domain waveform preservation and wavelet-domain time–frequency structural recovery during parameter optimization. Based on multi-fidelity robust Bayesian optimization, a unified robust parameter set suitable for the magnetic anomaly signal family is obtained.

Simulation experiments with colored noise and experiments using measured geomagnetic noise demonstrate that the proposed method can effectively suppress background noise and recover the main time-domain features of magnetic anomaly signals under low-SNR conditions. Compared with OBF, EMD, and CEEMDAN, the proposed method achieves better overall performance in terms of the correlation coefficient, wavelet-domain image structural similarity index, and detection probability. These results indicate that MPC-MDBSR has good applicability and robustness for low-SNR magnetic anomaly signal detection.

Nevertheless, the proposed method still has several limitations.

First, MPC-MDBSR generates a unified set of general optimization parameters for the magnetic anomaly signal family to ensure effective detection of signals with different morphological features. However, this robust parameter compromise may also limit the improvement in correlation performance for a specific waveform.

Second, although MPC-MDBSR can recover the main time-domain features of magnetic anomaly signals, it is mainly intended for detection and feature enhancement and does not directly provide a quantitative reconstruction of the true magnetic anomaly amplitude. Future work will focus on adaptive parameter optimization and quantitative magnetic anomaly amplitude recovery to improve the detection and characterization capability of the method in complex practical scenarios.

## Figures and Tables

**Table 1:** Main specifications of the TMR9082.

Supply Voltage	Sensitivity	Noise Spectral Density
1.5 V	100 mV/V/Gs	250 pT/rt(Hz)@1 Hz

## Data Availability

The data are not publicly available, due to the ongoing research.

## References

[1] Liu H., Zhang X., Dong H., Liu Z., Hu X. (2023). Theories, applications, and expectations for magnetic anomaly detection technology: A review. IEEE Sens. J..

[2] Gao J., Wang J., Zhang L., Yu Q., Huang Y., Shen Y. (2019). Magnetic signature analysis for smart security system based on TMR magnetic sensor array. IEEE Sens. J..

[3] Zhao J.-W., Zeng Z.-F., Zhou S., Guo H., Yan J.-H., Liu T.-Y. (2023). CWT-based magnetic anomaly data denoising method combining stochastic resonance system and pixel connectivity thresholding. IEEE Trans. Instrum. Meas..

[4] Billings S.D. (2004). Discrimination and classification of buried unexploded ordnance using magnetometry. IEEE Trans. Geosci. Remote Sens..

[5] Yuan Z., Liu Y., Xiang M., Gao Y., Suo Y., Ye M., Zhai Y. (2023). Compact multi-channel optically pumped magnetometer for bio-magnetic field imaging. Opt. Laser Technol..

[6] Ginzburg B., Frumkis L., Kaplan B.-Z. (2002). Processing of magnetic scalar gradiometer signals using orthonormalized functions. Sens. Actuators A Phys..

[7] Ginzburg B., Frumkis L., Kaplan B.-Z. (2004). An efficient method for processing scalar magnetic gradiometer signals. Sens. Actuators A Phys..

[8] Zalevsky Z., Bregman Y., Salomonski N., Zafrir H. (2012). Resolution enhanced magnetic sensing system for wide coverage real time UXO detection. J. Appl. Geophys..

[9] Sheinker A., Moldwin M.B. (2016). Magnetic anomaly detection (MAD) of ferromagnetic pipelines using principal component analysis (PCA). Meas. Sci. Technol..

[10] Sheinker A., Salomonski N., Ginzburg B., Frumkis L., Kaplan B.-Z. (2008). Magnetic anomaly detection using entropy filter. Meas. Sci. Technol..

[11] Sheinker A., Ginzburg B., Salomonski N., Dickstein P.A., Frumkis L., Kaplan B.-Z. (2011). Magnetic anomaly detection using high-order crossing method. IEEE Trans. Geosci. Remote Sens..

[12] Fan L., Hu H., Zhang X., Wang H., Kang C. (2022). Magnetic anomaly detection using one-dimensional convolutional neural network with multi-feature fusion. IEEE Sens. J..

[13] Sun T., Wang X., Wang J., Yang X., Meng T., Shuai Y., Chen Y. (2022). Magnetic anomaly detection of adjacent parallel pipelines using deep learning neural networks. Comput. Geosci..

[14] Benzi R., Sutera A., Vulpiani A. (1981). The mechanism of stochastic resonance. J. Phys. A Math. Gen..

[15] Wan C., Pan M., Zhang Q., Wu F., Pan L., Sun X. (2018). Magnetic anomaly detection based on stochastic resonance. Sens. Actuators A Phys..

[16] Zhang G., Tan C., He L. (2021). Piecewise unsaturated under-damped tri-stable stochastic resonance system and its application in bearing fault diagnosis. J. Vib. Eng. Technol..

[17] Cui W., Jiao S., Gao R., Zhang Q., Wang C., Li Y., Zhang Y. (2025). Continuous unsaturated second-order hybrid multi-stable stochastic energy resonance and its application in rolling bearing fault diagnosis. Appl. Acoust..

[18] Sun H., Qiu J., Huang S., Cao C., Zeng X. (2025). Magnetic anomaly signal processing method based on periodic potential stochastic resonance system. Measurement.

[19] Evstigneev M., Reimann P., Pankov V., Prince R. (2004). Stochastic resonance in monostable overdamped systems. EPL (Europhys. Lett.).

[20] Qin T., Zhou L., Chen S., Chen Z. (2022). The novel method of magnetic anomaly recognition based on the fourth order aperiodic stochastic resonance. IEEE Sens. J..

[21] Xu Z., Wang Z., Yang J., Sanjuán M.A., Sun B., Huang S. (2023). Aperiodic stochastic resonance in a biased monostable system excited by different weak aperiodic pulse signals and strong noise. Eur. Phys. J. Plus.

[22] Liu W., Liu Z., Zhang Q., Xu Y., Liu S., Chen Z., Zhu C., Wang Z., Pan M., Hu J. (2020). Magnetic anomaly signal detection using parallel monostable stochastic resonance system. IEEE Access.

[23] Kang Y.-M., Xu J.-X., Xie Y. (2003). Relaxation rate and stochastic resonance of a single-mode nonlinear optical system. Acta Phys. Sin..

[24] Wan C., Pang H., Mou S., Li H., Pan M., Zhang Q., Yang D. (2022). Magnetic anomaly detection using a parallel stochastic resonance system. IEEE Trans. Instrum. Meas..

[25] Sheinker A., Shkalim A., Salomonski N., Ginzburg B., Frumkis L., Kaplan B.-Z. (2007). Processing of a scalar magnetometer signal contaminated by 1/fα noise. Sens. Actuators A Phys..

